# Physiological Cues Involved in the Regulation of Adhesion Mechanisms in Hematopoietic Stem Cell Fate Decision

**DOI:** 10.3389/fcell.2020.00611

**Published:** 2020-07-10

**Authors:** Rohan Kulkarni, Vaijayanti Kale

**Affiliations:** ^1^The Ohio State University Comprehensive Cancer Center, Columbus, OH, United States; ^2^Symbiosis Centre for Stem Cell Research, Symbiosis International University, Pune, India

**Keywords:** physiological cues, hematopoietic stem cells, fate decision, adhesion mechanisms, ECM molecules, extra-cellular vesicles, HSC niche, stromal cells

## Abstract

Hematopoietic stem cells (HSC) could have several fates in the body; viz. self-renewal, differentiation, migration, quiescence, and apoptosis. These fate decisions play a crucial role in maintaining homeostasis and critically depend on the interaction of the HSCs with their micro-environmental constituents. However, the physiological cues promoting these interactions *in vivo* have not been identified to a great extent. Intense research using various *in vitro* and *in vivo* models is going on in various laboratories to understand the mechanisms involved in these interactions, as understanding of these mechanistic would greatly help in improving clinical transplantations. However, though these elegant studies have identified the molecular interactions involved in the process, harnessing these interactions to the recipients’ benefit would ultimately depend on manipulation of environmental cues initiating them *in vivo*: hence, these need to be identified at the earliest. HSCs reside in the bone marrow, which is a very complex tissue comprising of various types of stromal cells along with their secreted cytokines, extra-cellular matrix (ECM) molecules and extra-cellular vesicles (EVs). These components control the HSC fate decision through direct cell–cell interactions – mediated via various types of adhesion molecules –, cell-ECM interactions – mediated mostly via integrins –, or through soluble mediators like cytokines and EVs. This could be a very dynamic process involving multiple transient interactions acting concurrently or sequentially, and the adhesion molecules involved in various fate determining situations could be different. If the switch mechanisms governing these dynamic states *in vivo* are identified, they could be harnessed for the development of novel therapeutics. Here, in addition to reviewing the adhesion molecules involved in the regulation of HSCs, we also touch upon recent advances in our understanding of the physiological cues known to initiate specific adhesive interactions of HSCs with the marrow stromal cells or ECM molecules and EVs secreted by them.

## Graphical Abstract

Hematopoietic stem cells (HSCs) reside in the bone marrow microenvironment comprising of various types of stromal cells and the ECM molecules secreted by them. This complex microenvironment forms the specialized HSC niche, which controls their maintenance, survival and fate decisions. The HSCs interact and adhere to various niche cells such as MSCs, osteoblasts and ECs as well as with the ECM molecules secreted by them. The graphical abstract illustrates various molecules that have been shown to be expressed on the HSCs as well as stromal cells such as various integrins, N- cadherin, Notch, Ephrins, PSGL-1, CD162, CD44, etc. The image also shows that bone marrow microenvironment also contains extra-cellular vesicles (EVs, both micro-vesicles and exosomes) having an important role in HSC adhesion. The illustration also points out an as yet unexplored aspect of adhesive interactions between various stromal cells (e.g., between MSC-OB, MSC-EC etc., shown in dotted red lines).

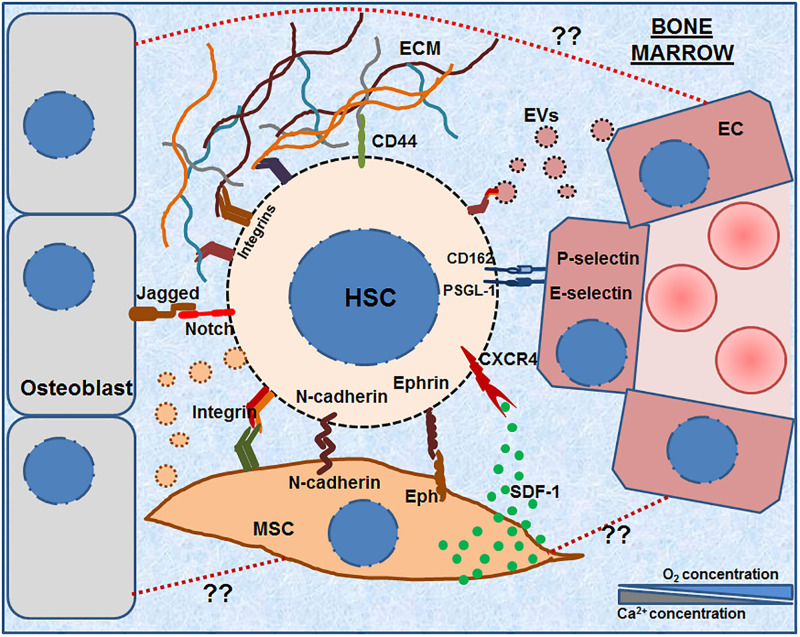

## Introduction

A hematopoietic stem cell (HSC) could have several fates in the body: it could remain quiescent; it could self-renew; it could commit to a particular lineage; it could migrate in response to a chemotactic cue; or it could even undergo apoptosis. In an adult human being the HSCs reside in bone marrow micro-environment comprising of several types of stromal cells along with their secretome. These micro-environmental components regulate or govern the HSC fate via signaling through various types of adhesion mechanisms. Since some of these mechanisms appear to involve short-range signals, HSCs need to be physically associated to the stromal cells: the adhesion molecules serve as the anchors that hold the HSCs in their niche. Although several studies have documented the adhesion mechanisms involved in the HSC fate decisions, the physiological cues that are translated into cellular language for the initiation of specific adhesive interactions of HSCs with stromal cells, or extra-cellular matrix (ECM) molecules and extra-cellular vesicles (EVs) secreted by them are not described to a great extent. Identification of such environmental cues could improve the outcome of clinical transplantations, as they can be harnessed to accrue benefit for the patients. Hence, in addition to reviewing the adhesion mechanisms operative in hematopoietic system, we also touch upon these aspects while describing them.

## Complexity of the HSC Niche

Hematopoietic stem cells are known to interact with a variety of cell types and extracellular components located in their close proximity. These interactions are crucially required for not only maintaining the stemness and the functionality of the HSCs, but also for governing their fate decisions. Schofield (1978) was the first person to coin the term “HSC Niche” while referring to the specific microenvironment present in the bone marrow which supports HSC maintenance and self-renewal. The present day concept of HSC niche involves various cell types, extracellular signaling molecules, ECM molecules, physical factors, etc. (Wang and Wagers, 2011). Recently, the EVs present in the bone marrow microenvironment secreted either by the marrow stromal cells or by the hematopoietic cells themselves have been identified as an important regulator of HSC adhesion (Butler et al., 2018).

Hematopoietic stem cells niches are thoroughly studied and characterized in invertebrate organisms such as *Caenorhabditis elegans* (Kimble and White, 1981) and *Drosophila melanogaster* (Xie and Spradling, 2000). Mammalian system is far too complex for such detailed and conclusive analyses. However, recent studies on mammalian systems have helped us to understand the indispensable role of the niche in governing the stem cell functionality. Development of several novel and sophisticated techniques such as real time imaging of cells have opened up new arena for understanding HSC and HSC niche biology. Picture of HSC niche is now becoming more explicit and the role of different niche components is now becoming a lot more comprehensive. The undifferentiated, long-term repopulating HSCs (LT-HSCs) are located near the bone endosteum and move in the direction of the central axis of the bone marrow in response to the mobilization or commitment signals (Lord et al., 1975; Gong, 1978). This niche, known as the endosteal niche, mainly contains pre-osteoblasts (Osteo-MSCs), osteoblasts and osteoclasts (Askmyr et al., 2009). Imaging of LT-HSCs for their spatial distribution confirms their presence in the endosteal zone of bone marrow (Zhang et al., 2003). The studies on HSC homing show that the infused HSCs home near the osteoblasts present in the endosteal niche in about 15 h after transplantation (Nilsson et al., 2001). Similar studies also suggest that the HSCs reside within about 200 μm of the sinusoidal blood vessel lining in the trabecular region of bone marrow cavity (Bourke et al., 2009). The histochemical studies of SLAM HSCs also reveal that majority of them are present in the close proximity of sinusoidal endothelial cells. This has led to the identification of second type of HSC niche termed as the perivascular niche (Kiel et al., 2005). The components of perivascular niche are mainly endothelial cells (ECs), mesenchymal stem cells (MSCs), cytokines, chemokine (C-X-C) ligand 12 (CXCL12)-abundant reticular (CAR) cells, platelet-derived growth factor receptor-α-expressing MSCs (PDGFR^+^ MSCs), Nestin positive MSCs, Macrophages, etc.

Bone marrow (BM) is a very complex structure made up of a variety of cell types having specific spatial locations (Beerman et al., 2017; Pinho and Frenette, 2019). The constantly changing dynamics of cellularity, blood perfusion and gradient of oxygen tension further adds to its complexity. A single HSC is known to receive and respond to a variety of signals emanating from the several types of niche cells simultaneously. Latest findings also show that the HSCs, though being present in their specific niches, can have cross talk with the long distance cells, which can modulate their functionality and decide their fate. These findings are now challenging the idea of anatomically distinct HSC niches and postulate that the entire bone marrow itself can be considered as a single niche, where discrete areas in the bone marrow compartment and the cell types present therein play important roles at different stages of hematopoiesis and co-ordinate the HSC maintenance, self-renewal, and differentiation (Wang and Wagers, 2011). Thus, the molecular understanding of mechanisms involved in HSC-niche interactions/adhesions mediating the cellular cross-talk still remains one of the most important areas of research in the field.

In the embryonic developmental stages, the HSCs are known to mobilize, migrate and home to various HSC niches in a coordinated manner. For example, HSC pool is known to move from yolk sac to fetal liver, from where it moves to thymus, to spleen and finally to the bone marrow just before the birth. These processes are governed by the adhesion molecules expressed on the HSCs during these migratory processes. These adhesion molecules also play an important role in migration and homing of the donor HSCs to the recipient’s bone marrow niche after transplantation. Considering their importance in clinical transplantations, the adhesion molecules are mostly studied either in the context of mobilization of the donor HSCs or their engraftment in the recipients’ marrow: the processes working in exactly opposite direction of each other. Various adhesion molecules (Table 1) that are expressed on the HSCs and their niche cells are discussed below and are illustrated in the Graphical Abstract.

**TABLE 1:** Summary of adhesion molecules expressed on hematopoietic stem cells and their counter receptors on niche components.

Sr. no.	Adhesion molecules on HSCs	Receptor molecules in niche	Niche component expressing receptor molecules	References
1	Integrin β1αL (LFA-1)	ICAM-1	Endothelial cells	Shimizu et al., 1990
2	Integrin β1α4 (VLA-4)	VCAM-1/fibronectin	Endothelial cells	Peled et al., 2000
			ECM	Shimizu et al., 1990
3	Integrin β1α45 (VLA-5)	Fibronectin	ECM	Shimizu et al., 1990
4	Integrin β1α6 (VLA-6)	Laminin	ECM	Shimizu et al., 1990
5	Integrin β1α2	Fibronectin Fibrinogen Vitronectin von Willebrand factor	ECM	Gekas and Graf, 2013
6	Integrin β1α9	VCAM-1	Endothelial cells	Liu et al., 2018
7	Integrin β3α5	MAdCAM Periostin	ECM	Khurana et al., 2016
8	Integrin β7α4	VCAM-1	Endothelial cells	Katayama et al., 2004
9	ESL-1	E-selectin	Endothelial cells	Leiva et al., 2016
10	PSGL-1	E-selectin	Endothelial cells	Leiva et al., 2016
		P-selectin	Endothelial cells	Lévesque et al., 1999
11	CD162	P-selectin	Endothelial cells	Lévesque et al., 1999
12	CD34	L-selectin		
13	CD44	Hyaluronic acid	MSC ECM	Avigdor et al., 2004; Wagner et al., 2008
14	Notch	Jagged	MSCs Endothelial cells Osteoblasts Osteoclasts	Azizidoost et al., 2015
15	N-cadherin	N-cadherin	MSCs	Zhao et al., 2019
16	Eph	Ephrin	MSCs	Foo et al., 2006

## Important Adhesion Molecules Involved in HSC Adhesion

### Integrins

Integrins are one of the most important classes of adhesion molecules involved in the interaction of HSCs with their micro-environment. They not only participate in cell–cell and cell–matrix interactions, but also make connections to the cytoskeleton through their cytoplasmic domains and activate many intra-cellular pathways. Most integrins interact with several different ECM proteins, and individual matrix proteins bind to several integrins (Hynes, 2002). Owing to their characteristic bidirectional signaling mechanism, coupled with their ability to interact with various growth factor receptors, integrins are able to coordinate signals from the growth factors and the ECM molecules to support cell proliferation, migration, etc. (Eliceiri, 2001).

Integrins are made up of α and β subunits that are non-covalently associated with each other (Hynes, 2002). There are mainly 24 types of integrins formed from a combination of one α- and one β-subunits from 18 types of α- and 8 types of β-subunits (Takada et al., 2007). Integrins are classified on the basis of their constituting β-subunits. These integrins bind to various types of ECM molecules like fibronectin, vitronectin, laminins, fibrinogens, collagens as well as the complimentary receptors expressed on neighboring cells. In addition to this, integrins play an important role in providing the mechanical link between ECM and the cellular cytoskeleton. Moreover, their role as the receptors for many signaling molecules and cytokines is quite well known (Barczyk et al., 2010).

In the hematopoietic system, integrins are known to regulate important functions such as cell signaling, adhesion, migration and homing. The process of migration of HSCs to the specific niches in the bone marrow compartments after their transplantation is known as HSC homing. HSCs are known to express β1, β2, β3, and β7 integrins. Most common and widely studied ones is the β1 integrin, which dimerizes with α1, α2, α4, α5, and α6 to form various types of functional adhesion molecules (Levesque and Winkler, 2016). The importance of the β1 integrin in HSC migration and homing was evident by the fact that the β1 integrin knock-out embryos die during the embryogenesis (Hirsch et al., 1996). HSCs from β1^–/–^ chimeric embryos were unable to migrate to fetal liver marking the crucial role of β1 integrins in HSC migration and homing. Similarly, the conditional β1 knockout HSCs fail to migrate to hematopoietic organs such as bone marrow, spleen and thymus (Potocnik et al., 2000). Changes in the expression levels of integrin molecules have been shown to be associated with mobilization as well as homing and engraftment of HSCs. The Cyclophosphamide/Granulocyte- Colony Stimulating Factor (G-CSF) mobilized HSCs collected from peripheral blood were seen to have a down-regulated expression of integrins and these HSCs were compromised in their homing and engraftment ability. Pre-treatment of HSCs with blocking monoclonal antibody against α4 integrin function results in their decreased homing (Wagers et al., 2002). Specifically, α4β1 (VLA-4), α5β1 (VLA-5), and αLβ2 (LFA-1) are important for HSC-EC adhesion and the subsequent *trans*-endothelial migration of HSCs toward the stromal cell-derived factor (SDF)-1α (CXCL12)-expressing stromal cells (Peled et al., 2000). Blocking of α5β1 integrins on HSCs before transplantation significantly reduces their homing to bone marrow (Wierenga et al., 2006). Along with the above-mentioned integrin molecules, α4β7 integrin has also been found to play a role in post-transplantation recruitment of HSCs into bone marrow (Katayama et al., 2004). Integrin α6β1, known as CD49f, is also expressed on hematopoietic cell population and is known to regulate the homing of a specific subset of HSCs having long term multi-lineage engraftment potential (Notta et al., 2011). It is currently used as a marker for distinguishing HSCs from multi-potent progenitors (MPPs) in human hematopoietic system. Yet another integrin that plays an important role in HSC functions is αvβ3. Thrombopoietin-mediated maintenance of HSCs depends on signaling emitted by activated αvβ3 integrin (Umemoto et al., 2012). Khurana et al. (2016) demonstrated that Periostin (POSTIN), an ECM molecule, interacts with αvβ3 and regulates HSC proliferation by increasing the expression of p27Kip1 (cdkn1b). Loss of POSTIN leads to HSC proliferation coupled with loss of their functionality.

Integrin αIIbβ3 (CD41/CD61) is known to participate in cell adhesion and cell surface-mediated signaling. Its expression is mostly restricted to megakaryocytes, but a small subset of HSCs also expresses CD41. In the embryonic hematopoietic cells, CD41 is considered to be the marker for differentiation. Its expression has been shown to be associated with early stages of hematopoiesis and is tightly regulated during hematopoietic development (Mitjavila-Garcia et al., 2002). Adult HSCs also have been shown to express CD41 on their surface. These CD41^+^ HSCs accumulate during aging. They are found to be largely quiescent and exhibit myeloid bias. They also show myelo-erythroid and megakaryocyte gene priming, which is governed by Gata1 (Gekas and Graf, 2013).

#### Integrins as Regulators of HSC Adhesion in Response to Matrix Stiffness

An aspect that is now fast emerging is how HSCs use various adhesion molecules and receptors to respond to the changes in the stiffness of the microenvironment; integrins play a crucial role in this process. The integrin-mediated adhesion starts with initial attachment of the cells to the matrix, where integrin clustering, recruitment of adhesion-associated proteins, and the generation of focal adhesion complexes take place (Wolfenson et al., 2013). This ability of integrins to recruit adhesion-associated proteins helps them to sense and respond to different kinds of extracellular biophysical cues of the microenvironment.

Biophysical cues such as matrix stiffness and mechanical force need critical consideration while developing matrices for stem cell maintenance and growth *in vitro* (Choi et al., 2015). In addition to providing biological cues, the ECM molecules also provide biophysical cues via various adhesive mechanisms (Zhang et al., 2019). HSCs respond to the external biophysical signals by binding to ligands present on the adhesive substrates through clustering of various adhesion molecules including integrins, which initiates transduction of “outside-in” signaling in them. The bone marrow microenvironment provides a gradient of varying stiffness, which affects the HSC fate. The endosteal region of the bone is relatively stiff having Young’s modulus of 40–50 kPa, as compared to the perivascular region having a modulus of about 3 kPa and the central medullary region having a modulus of 0.3 kPa (Greenbaum et al., 2013; Choi and Harley, 2017). Since endosteal niche harbors quiescent HSCs having long-term engraftment ability, while perivascular niche harbors actively proliferating HSCs having short-term engraftment potential, it appears that that perhaps matrix stiffness contributes in controlling the quiescence and the functionality of HSCs. Taking a cue from the *in vivo* observations, Chitteti et al. (2015) cultured mouse bone-marrow derived LSK HSCs in 3D matrix created using varying concentrations of type I collagen oligomers giving varying stiffness (50–800 Pa) and calvariae-derived osteoblasts. They found that indeed the decreased proliferation of LSK HSCs was associated with an increase in the matrix stiffness. LSK HSCs present within 800 Pa collagen oligomer matrices maintained the highest percentage of cells in quiescent state (G0/G1), as compared to those present within 50 Pa or 200 Pa matrices. Lee-Thedieck et al. (2012) demonstrated that osteoblasts flatten and remodel their cytoskeleton in response to the adrenergic agonist clenbuterol. These changes increase the stiffness of the matrix, which enhances HSC adhesion and migration (Lee-Thedieck et al., 2012). These findings could have implications in HSC mobilization. Matrix stiffness also determines the commitment process of the HSCs. Using ECM-coated polyacrylamide surfaces of varying stiffness Choi and Harley (2017) showed that stiffer substrates (44 kPa) promote higher formation of CFU-GEMM (Colony Forming Unit- Granulocyte-Erythrocyte-Monocyte-Megakaryocyte), CFU-E and CFU-M (Macrophage) type of colonies, whereas softer substrates (3.7 kPa) support formation of CFU-G (Granulocyte).

### Selectins

Selectins play a very important part in the homing of transplanted HSCs to the bone marrow niche. Due to the rapid association and dissociation rate constants that characterize the adhesion mediated by them, selectins play an important role in tethering and rolling of transplanted HSCs along the walls of endothelial capillaries. Selectins recognize fucosylated, sialylated, and sulfated ligands expressed on scaffold glycoproteins serving as functional counter-receptors. They are regulated at the transcriptional level, through various mechanisms such as proteolytic processing, cellular sorting, and regulated expression of glycosyl-transferases responsible for the formation of functional ligands. Each selectin molecule consists of an extracellular lectin-like domain, an EGF-like domain, and variable numbers of a consensus repeat bearing homology to complement proteins, followed by transmembrane and short intracellular sequences.

Selectin family consists of three calcium-dependent lectins viz. E-selectin, P-selectin, and L-selectin. They are involved in the recruitment of leukocytes to the sites of inflammation (Kansas and Pavalko, 1996).

#### E-Selectin

Amongst the three selectins, E-selectin has been shown to play a unique role in HSC adhesion. Under steady state conditions, very few vascular ECs present in the bone marrow express E-selectin. However, post-irradiation more than 90% of them express E-selectin and this promotes a rapid proliferation of HSCs. Consistent with this, a therapeutic blocking of E-selectin or genetic deletion of E-selectin promotes HSC quiescence (Winkler et al., 2012). This approach can be applied when HSCs need to be protected during aggressive chemotherapy. Both, human and mouse HSCs express E-selectin ligands namely, PSGL-1 and CD44, but the anti-proliferative effect of E-selectin is not seen in PSGL-1 or CD44 KO mice, suggesting involvement of yet another un-identified ligand involved in E-selectin-mediated HSC quiescence. E-selectin ligand 1 (ESL-1) is expressed by primitive HSCs. Deletion of ESL-1 leads to HSC quiescence, but this effect is E-selectin-independent (Leiva et al., 2016); rather, this quiescence was due to higher secretion of TGFβ1 by the mutant HSCs. Such findings throw light on complexity of the adhesion molecules and their effects of HSC fate.

#### P-Selectin

P-selectin is typically found on the surface of activated ECs and platelets. It is reported to play an important role in recruitment of lymphocytes at the site of inflammation. In response to inflammatory signals, P-selectin gets localized on the surface of platelets and stimulates the ECs (Chen and Geng, 2006). Pro-inflammatory cytokine TNF-α is known to induce the expression of P-selectin in the ECs (Hahne et al., 1993). P-selectin molecules are stored in the form of granules inside the cells until the signal for its surface expression is received from extracellular pro-inflammatory signaling molecules like TNF-α (Vestweber and Blanks, 1999). The inhibitors of phosphatidylinositol 3 kinases (PI3K), Wartmanin and LY294002, are reported to abrogate the secretion of P-selectin granules in platelets (Flaumenhaft et al., 2005). RANTES, yet another pro-inflammatory cytokine, is reported to trigger the recruitment of monocytes to the endothelium during inflammation (Gawaz et al., 2005). Transplantation of wild type HSCs fail to home in the bone marrow compartment of lethally irradiated P-selectin^–/–^ recipient mice, confirming the indispensable role of P-selectin in the homing of HSCs to their niche (Frenette et al., 1996). LT-HSCs are reported to express CD162, the receptor for P-selectin. Culturing of LSK (Lin^–^Sca-1^+^c-Kit^+^) cells on immobilized P-selectin molecules delays the differentiation process and promotes their proliferation resulting in increased CFU-S and CFC numbers in *in vivo* mouse models (Eto et al., 2005). P-selectin is also known to negatively regulate hematopoietic progenitors via its binding to CD162 (Lévesque et al., 1999). Recent reports show that P-selectin is also expressed on primitive HSC population. The P-selectin expression was documented to be increased in the aged HSCs due to increased inflammation-like signaling in aged HSC niche (Chambers et al., 2007). Another recent study done using quantitative mass spectrometry analysis of expressed proteins confirms the significantly higher expression of P-selectin in the HSC populations upon induction of acute inflammation, mimicking the viral infection in the mice injected with polyinosinic-polycytidylic acid (Haas et al., 2015).

Collectively, these reports show that inflammation, induced by pre-transplant conditioning regimes, infections, or aging, appears to be one of the physiological cues regulating E- and P-selectin expression on the HSCs and the ECs. However, how the inflammatory conditions lead to an increase in calcium levels required for the activation of these selectins remains to be examined.

#### L-Selectin

L-selectin is known to bind to P-selectin glycoprotein ligand-1 (PSGL-1) and a specialized glycoform of CD44 (hematopoietic cell E- and L-selectin ligand, HCELL). Although human hematopoietic cells are shown to interact with both the molecules, under shear stress condition, human hematopoietic cells adhere strongly to HCELL, as compared to PSGL-1 (Dimitroff et al., 2001). This L-selectin ligand activity of HCELL requires sialofucosylated N-linked glycans and is sulfation independent (Dimitroff et al., 2000, 2001). Earlier, we have shown that the cryopreservation of CD34^+^ human HSPCs in the presence of interleukin-3 (IL-3) and stem cell factor (SCF) increases the frequency of CD34 and L-selectin double positive HSCs in the revived cell population, along with an increased number of CFU-forming progenitors (Sasnoor et al., 2003), indicating the importance of L-selectin expression on human HSPCs. Although L-selectin (CD62L) is expressed on HSCs and HPCs, it role in HSC adhesion remains unclear (Levesque and Winkler, 2016). It has been shown to be expressed on mobilized human CD34^+^ cells (Möhle et al., 1995) and murine HSCs (Mendez-Ferrer and Frenette, 2007). However, L-selectin does not participate in the interaction of murine HSCs with bone marrow microvessels (Mazo et al., 1998), but the expression of L-selectin was observed to be reduced on G-CSF mobilized human CD34^+^ cells 4 and 6 days post-mobilization (Bellucci et al., 1999). Gunji et al. (1992) showed that incubation of human HSPCs *in vitro* with anti-L-selectin antibody blocked clonogenic outgrowth of cells in both long-term and short-term (methylcellulose) assays (Gunji et al., 1992). A progressive upregulation of L-selectin (along with other 5 genes, namely Col4a1, Entpd1, Mmp2, Tgfbi, and Timp2) was seen in fetal liver LSK cells at 14.5 dpc (Ciriza and García-Ojeda, 2010), suggesting that perhaps these genes play an important role in the HSCs during development, possibly in their migration from fetal liver to the bone marrow. Koenig et al. (1999) found that a higher proportion of CD34^+^ HSCs from cord blood express L-selectin, as compared with those from adult bone marrow. They further showed that the CD34^+^ cord blood HSCs expressing L-selectin form a significantly higher number of colonies, as compared to those lacking L-selectin expression.

Interestingly, L-selectin appears to have a role in the adhesion of leukemic stem/progenitor cells. Krause et al. (2014) demonstrated that BCR-ABL1–expressing L-selectin–deficient progenitors could not induce CML-like leukemia after i.v. injection into wild type recipients. Their findings could be harnessed in preventing re-engraftment of Ph+ (Philadelphia Chromosome Positive) leukemic stem cells in CML patients treated by autologous transplantation. Expression of L-selectin was seen to be progressively increasing on the AML blast cells with age. This increased expression correlated with decreased apoptosis of blast cells upon treatment with cytarabine arabinoside (Ara-C). Thus, disruption of L-selectin – bone marrow microenvironment interaction can be used as a strategy for sensitizing the leukemic cells for chemotherapy (Huang et al., 2016).

### N-Cadherin

Role of N-cadherin in the regulation of HSC fate is by far the most controversial matter in the history of adhesion molecules involved in the process. Expression of N-cadherin on HSCs became a matter of hot debate as one group strongly advocated the expression of N-cadherin on the HSCs and its role in the regulation of hematopoiesis (Calvi et al., 2003; Zhang et al., 2003; Arai et al., 2004; Wilson et al., 2004; Hosokawa et al., 2007, 2010a; Haug et al., 2008), whereas another group vehemently denied the same (Kiel et al., 2007, 2009). To resolve the controversy, a meeting with the principal investigators was arranged (Li and Zon, 2010). While the questions related to the expression of N-cadherin on HSCs and the antibodies to be used to detect the same were clarified, whether N-cadherin plays a role in HSC maintenance and regulation could not be resolved at that time. Overall conclusion regarding the first two points was that “*N-cadherin is expressed at extremely low levels in a very limited hematopoietic cell population*,*or may in fact be absent*,” and “*the N-cadherin monoclonal antibody*,*MNCD2*,*is not specific for N-cadherin expression in the hematopoietic compartment.*” Whether such low level expression of N-cadherin on the HSCs as reported by the investigators translates into meaningful regulatory role is still an open question.

Notwithstanding this controversy, the unresolved matter related to the role of N-cadherin in HSC fate determination is still under active investigation in several labs. Using single cell gene expression analysis Arai et al. (2012) showed the presence of N-cadherin (*Cdh2*) in the HSCs (LSK CD150^+^ CD34^–^), albeit at a variable level. Using a newly developed antibody to N-cadherin they also showed that the LSK HSCs express N-cadherin and the N-cadherin^+^ HSCs possess higher engraftment ability. On the background of previous controversy over the N-cadherin antibody (MNCD2), these data need confirmation from other labs. The same group (Hosokawa et al., 2010b; Arai et al., 2012) showed that shRNA-mediated silencing of N-cadherin in the HSCs increases their proliferation and significantly affects their long-term reconstituting ability.

As against the expression of N-cadherin in HSCs, there appears to be consensus over the expression of N-cadherin in the stromal cells, but whether it plays any role in the regulation of HSC functions remains controversial. Using genetic manipulation of Cdh2, a gene encoding N-cadherin, two groups demonstrated that deletion of N-cadherin from osteoblastic cells, both, osteoprogenitors and more mature osteoblasts, had no effect on steady state hematopoiesis or on any of the HSC functions such as engraftment, proliferation or mobilization (Bromberg et al., 2012; Greenbaum et al., 2012; Levesque, 2012). These data showed that N-cadherin does not play any regulatory role on the HSCs in the osteoblastic niche. On the other hand, using a chemotherapy model, Zhao et al. (2019) recently identified two classes of HSCs that exist in the BM niche; one, reserved HSCs (rHSCs, CD48^–^CD49b^–^ LT-HSCs), and the other, primed HSCs (pHSCs, CD48^–^CD49b^+^ LT-HSCs), and showed that the N-cadherin^+^ bone and marrow progenitor cells (BMSPCs) play an important role in protecting rHSCs from chemotherapy. They further found that genetic ablation of N-cadherin from BMSPCs impaired rHSC maintenance during homeostasis and regeneration. These data show that N-cadherin positive stromal cells differentially regulate HSC subsets.

In contrast to the murine data, an early study by Puch et al. (2001) showed that N-cadherin is expressed on a subpopulation of human CD34^+^ HSCs and also on bone marrow stromal cells. A function blocking antibody to N-cadherin diminished the colony formation by the HSCs and also interfered with the adhesion of KG1a cells, a CD34^+^ cell line of leukemia origin, to stromal cells. Wein et al. (2010) showed that N-cadherin was expressed at moderate levels in CD34^+^ HSCs isolated from cord blood (CB) and mobilized peripheral blood (MPB). Cadherin-11 was expressed on CB HSCs only. Both adhesion molecules were strongly expressed on the MSCs. They also found co-localization of N-cadherin and β-catenin at the junction of HPC and MSC, which could be disrupted using siRNA-mediated knockdown of N-cadherin or cadherin-11 as well as treatment with a blocking antibody. Such intervention also impaired maintenance of long-term culture-initiating cells (LTC-IC) in the co-culture of HSCs and MSCs. These data showed that N-cadherin-mediated adhesive interactions are important for human HSCs. However, in this study they did not determine the *in vivo* effects of N-cadherin knock out. Reichert et al. (2015) reported a functional difference between the MSCs isolated from murine vs. human bone marrow in terms of their support to human HSCs *in vitro*. Using atomic force microscopy (AFM)-based single-cell force spectroscopy (SCFS), they showed that human HSCs have a stronger adhesion to human MSCs, as compared to their adhesion with murine MSCs. They correlated this difference to the N-cadherin levels in these MSCs. These data suggest that N-cadherin could have a species-specific role in the regulation of human HSCs. However, more rigorous *in vivo* studies are needed to come to a definitive conclusion.

In spite of several studies dealing with the role of N-cadherin in hematopoiesis, the physiological stimulus or cue regulating the N-cadherin expression on the HSCs or the niche cells has not been examined. In this context, using murine BMSCs we showed that hypoxia up-regulates expression of N-cadherin in them and this expression is very sensitive to oxygen (Moirangthem et al., 2015). Interestingly, we found that shRNA-mediated down-regulation of N-cadherin on stromal cells reduces the expression of CXCR4^+^ on the co-cultured HSCs, leading to a significant reduction in their chemotactic migration toward SDF1α. Such silencing of N-cadherin in the stromal cells results in an increased pool of HSCs, but the frequency of CXCR4^+^ HSCs goes down drastically. Based on our results, we speculated that perhaps the temporary normoxia that follows myeloablation as a consequence of reduction in the cell number perhaps leads to a transient down-regulation of N-cadherin in the BMSCs, thereby allowing the HSCs to proliferate. After HSC regeneration, hypoxia sets in due to high consumption of local oxygen and the expression of N-cadherin returns to the normal level. Although speculative, this study points toward the role of hypoxia as a physiological cue involved in the regulation of N-cadherin-mediated adhesion of HSCs with the BMSCs. This needs to be formally examined in *in vivo* situation.

### Notch Receptors

Notch is a cell-surface receptor that transduces short-range signals by interacting with transmembrane ligands such as Delta-like and Jagged on neighboring cells (Kopan and Ilagan, 2009; Kopan, 2012). In mammals, four Notch receptors (Notch 1–4) and five structurally related, single-pass membrane Notch ligands (Delta-like1, 3, and 4 and Jagged1 and 2) have been identified. After their synthesis, Notch receptors are cleaved by protein convertases followed by a second cleavage by the γ-secretase complex, and during their passage through Golgi they can be glycosylated by specific glycosyltransferases. Post-translational modifications determine the subsequent response to different ligands (Haines and Irvine, 2003).

Canonical Notch signaling is activated when Notch receptor binds to its ligand leading to its cleavage and release of the Notch intracellular domain (NICD), which then travels to the nucleus and binds to CSL [CBF1/RBPjk/Su(H)/Lag1]. CSL interacts with many other proteins to form either repressor complexes comprising histone deacetylases (HDACs), which preserve a closed chromatin conformation, or activating complexes containing NICD and histone acetyltransferases (HATs), which open up chromatin. The extracellular domain of all Notch receptors contains 29–36 tandem epidermal growth factor (EGF)-like repeats. Interestingly, when Notch receptors and their respective ligands are present on adjacent cells (in *trans*) the interaction becomes productive, whereas when they are present on the same cell (in *cis*), the activation is inhibited (Sprinzak et al., 2010). Productive interactions are mediated by repeats 11–12, whereas inhibitory interactions are mediated by repeats 24–29. The activation of Notch signaling triggers the expression of various target genes, such as Hes and the Hes-related (HESR/HEY) family of basic helix-loop-helix transcription repressors, which in turn regulate the expression of other genes.

Notch signaling plays an important role in the regulation of hematopoiesis starting from embryonic hematopoiesis to a definitive one (Butko et al., 2016). It has been shown to have various effects on HSCs, MSCs, osteoclasts, as well as osteoblasts (Azizidoost et al., 2015). Activation of Notch signaling requires a direct cell–cell contact through the interaction of Notch receptors (Notch 1–4) with their ligands (Jagged1–2 and Delta like 1, 3, and 4), and hence, it plays an important role in HSC adhesion (Baron, 2003). Immobilized Notch ligands can induce high levels of Notch activation in the cultured HSCs, and therefore, this approach has been used for their *ex vivo* manipulation (Varnum-Finney et al., 2000; Ohishi et al., 2002).

The presence of Notch ligands has been shown in various bone marrow stromal cells viz. ECs, osteoblasts, and MSCs. Fernandez et al. (2008) showed that *in vivo* pro-inflammatory cues like TNFα and LPS cause an increase in Jagged 2 expression on marrow ECs and Notch1 and Notch2 receptors on hematopoietic progenitors. Thus, like P-selectin the inflammatory conditions prevailing during pre-transplant myeloablation (Hill and Wu, 2009) could stimulate the expression of Notch1 and Notch2 receptors on the HSCs, thereby facilitating HSC engraftment and proliferation. Butler et al. showed that ECs support a long-term expansion of Notch-activated LSK HSCs, but not of the Notch1- and Notch2-deficient ones (Butler et al., 2010). Further, this group also showed that a conditional deletion of *Jagged1* in ECs causes a decrease in the number of HSCs in steady-state conditions. This reduction was due to a significant reduction of Notch activation in LSK HSCs (Poulos et al., 2013). Importantly, these Jagged-1 KO mice showed deficient hematopoietic recovery post-irradiation, underscoring the importance of Notch signaling activation by Jagged-1 expressed on bone marrow endothelial cells in maintaining both, homeostatic and regenerative hematopoiesis. Corselli et al. (2013) showed that a subset of human CD146^+^ perivascular MSCs expresses significantly higher levels of Jagged-1, as compared to the un-fractionated MSCs and CD146^–^ cells, and support long-term maintenance of functional human HSCs, a process partly meditated through Notch activation. Consistent with this report, we found that micro-vesicles (MVs) secreted by nitric oxide-primed BMSCs (primed MVs) contain significantly higher concentration of Jagged-1-specific mRNAs that upon transfer to the HSCs increase the level of Jagged-1 in them leading to their enhanced engraftment (Jalnapurkar et al., 2019). Parathyroid hormone (PTH) is known to increase the expression of Jagged-1 in the HSCs, leading to their expansion (Yamada et al., 2003; Wilson and Radtke, 2006). An interaction of Notch pathways with HoxB4 (homeobox B4) and Wnt signaling has been shown to support maintenance and self-renewal of HSC (Jacobsen, 2005; Chotinantakul and Leeanansaksiri, 2012). HSCs having self-renewal capacity tend to localize to the endosteal niche present in the trabecular bone area. These HSCs exhibit significantly higher level of Notch signaling activation, as compared to those present in the long bone area. Consistent with this activation profile, osteoblasts from the endosteal regions of the trabecular bone area show increased expression of Jagged-1, Jagged-2, and Delta-like4, as compared to the osteoblasts from long bone area (Guezguez et al., 2013). Collectively, these studies underscore the importance of Notch signaling in the retention of functional HSCs in the niche.

Knockdown of Notch signaling pathway, including prevention of the proteolytic cleavage of the intracellular domain of Notch, leads to a decrease in the repopulation of HSC pool (Jacobsen, 2005; Butler et al., 2010). Role of Notch signaling in HSC adhesion and niche retention was revealed by the study done by Wang W. et al. (2015). Using mice with conditional KO for *Pofut1* they demonstrated that *Pofut1*-deficient HSCs do not adhere well to Notch ligand-expressing marrow stromal cells lose their quiescent state and egress from the marrow. This was further confirmed by using neutralizing antibodies to DLL4 and JAG1. Deletion of Notch1 alone, or Notch1 and Notch2 together was found to increase the differentiation of lymphoid HSCs (Bigas and Espinosa, 2012). Wang et al. (2017) found that Notch 2, but not Notch 1, blockade leads to HSC mobilization. Interestingly, Notch 2 blocked, but not Notch 2 deficient, HSCs exhibited a competitive repopulating advantage and enhanced hematopoietic reconstitution. This study revealed distinct functions of two Notch receptors and suggested that this strategy can be applied to improve the outcome of clinical transplantations. Collectively, these studies underscore the importance of Notch signaling in HSC adhesion to the stromal components.

## Other Adhesion Molecules Involved in HSC Adhesion

In addition to the major players like N-cadherin, selectins, integrins, and Notch family members, several other adhesion molecules have been shown to play important role in adhesion, retention and engraftment of HSCs in their niche. Here we review some of the other molecules identified to play a role in HSC adhesion to various stromal cells or the ECM molecules secreted by them. However, in these studies also the physiological cues regulating the HSC adhesion have not been investigated.

### CD44

Although encoded by a single gene, CD44 has several isoforms arising due to both, alternate splicing and post-transcriptional modifications. CD44 standard (CD44s) is the most abundantly expressed isoform in mammalian cells. It binds to several ECM molecules, amongst which hyaluronan is the most common ligand. Hyaluronan is an important component of the HSC niche and participates in HSC lodgement in the endosteal region. CD44, hyaluronan and SDF-1 interaction has been shown to affect HSC and progenitor cell trafficking (Avigdor et al., 2004; Wagner et al., 2008). Using a CD44^–/–^ mouse model, Cao et al. (2016) showed that CD44 is critically required for the maintenance of the HSC pool, as well as in the HSC homing and lodgement into the bone marrow. Absence of CD44 brought down the % chimerism to less than 50%, but did not change the frequency of LT-HSCs. Likewise, a significantly higher number of donor WT HSCs was required to obtain 100% chimerism in CD44 KO recipient mice. Additionally, they showed that CD44 is also involved in the migration of fetal HSCs out of the liver, *via* a process involving SDF-1α. These studies underscore the importance of CD44 in the HSC adhesion.

Studies done on human HSCs also suggest the role of CD44 in their adhesion to niche components. Since adhesion molecules form the main target of HSC mobilization strategies, several investigators study the adhesion molecules in the context of mobilization. Szmigielska-Kaplon et al. (2014) analyzed the adhesion molecule expression on mobilized CD34^+^ HSCs and found that the CD44 TT genotype was the only factor associated with a higher risk of poor mobilization of HSCs in patients with hematological malignancies. Specifically, among the single nucleotide polymorphisms (SNPs) investigated in the study, only CD44 rs13347 was found to have an impact on the efficacy of HSCs mobilization, suggesting that analysis of CD44 SNPs may help in identifying the poor mobilizers. Such analysis could lead to the development of novel approaches of using inhibitors of adhesion molecules for HSC mobilization. Cecyn et al. (2018) examined the correlation of expression of various cell adhesion molecules on the yield of CD34^+^ HSCs in the peripheral blood after mobilization. They determined the correlation between various cell adhesion molecules (CAMs) with the yield of CD34^+^ cells and found that poor yield of CD34^+^ HSCs correlated with a higher expression of CD44, CD106, and CD49d, as compared with those with good CD34^+^ cell yield. Mobilized CD34^+^ HSCs showed a reduced expression of several CAMs like CD44, CD106, CD135, CD49d, and CD11a, but this did not correlate with the yield. These data show the importance of various adhesion molecules in the mobilization of HSCs required for clinical transplantations and suggest that alternate strategies involving targeting of these adhesion molecules might be required to be applied in case of poor mobilizers. Loeffler et al. (2018) used the HSC adhesive properties of CD44 and CD43 to make a technical advancement in the time-lapse microscopic studies on HSCs. They demonstrated that seeding of murine LSK HSCs or human CD34^+^ HSCs on plates coated with antibodies to CD44 and CD43 reduced their mobility and enabled colony formation in liquid cultures, increased imaging throughput in time-lapse microscopy, and also facilitated media change without affecting cell positions (Loeffler et al., 2018).

### SDF-1α/CXCR4 Axis

The success of HSC transplantation critically depends on the ability of transplanted HSCs to mobilize, home, migrate, and efficiently engraft in the bone marrow niche. SDF-1α (CXCL12)–CXCR4 axis plays a crucial role in the process (Bonig et al., 2006; Dar et al., 2006; Karpova et al., 2016). SDF1α provides a chemotactic cue to the transplanted HSCs, and once they engraft, it helps in the retention of the HSC via its interaction with CXCR4. Deletion of either Sdf1 or Cxcr4 is lethal in late embryonic stages, as these embryos lack bone marrow hematopoiesis (Nagasawa et al., 1996; Zou et al., 1998). However, deletion of Cxcr4 in adult HSCs does not affect their homing and engraftment, suggesting that other mechanisms compensate for its loss (Sugiyama et al., 2006; Nie et al., 2008).

Both, HSC retention and mobilization are largely governed by SDF-1α- CXCR4 axis and by α4-integrin signaling. Both pathways are dependent on c-kit activity. Inhibition of c-kit kinase affects HSC mobilization in response to either CXCR4 antagonists or α4-integrin blocking agents and also prevents the retention of CXCR4^+^ HSCs in the bone marrow.

Some adhesion molecules like endolyn (CD164) have been shown to impart specificity to the HSC-niche interactions mediated by the SDF1α-CXCR4 axis. Watt and Forde (2008) have shown that CD164, a member of sialomucin family of adhesion receptors, associates with CXCR4 and regulates the adhesion of CB-derived CD34^+^ cells to bone marrow stroma and promotes cycling of CD34^+^ human HSCs. They further showed that use of a function disrupting antibody or CD164 knock down by RNAi significantly inhibits the migration of CB-derived CD133^+^ HSCs toward SDF1α *in vitro*. They further showed that CD164 associates with CXCR4 in the presence of SDF1α and fibronectin, followed by the association of CXCR4 with the integrins VLA-4 and VLA-5. This interaction leading to the activation of PKC-zeta and Akt signaling gets disrupted in the absence of CD164. Pello et al. (2006) found that bovine growth hormone transgenic mice have a larger number of LSK HSCs having up-regulated SOCS1 and SOCS3 expression and impaired CXCL12(SDF1α)-induced function in their peripheral blood, as compared to control mice. CXCR4 expression was, however, not affected in these HSCs. This effect was recapitulated by exogenously provided growth hormone. These observations suggested a role for SOCS in controlling CXCL12-mediated HSC retention in bone marrow. Smith-Berdan et al. (2011) reported that Robo4^–/–^ HSCs display poor localization to bone marrow niches leading to poor engraftment. Interestingly, loss of Robo4 led to up-regulation of both Scf1 and Cxcr4, perhaps as a compensatory mechanism. Robo4 deletion also affects the efficiency of HSC mobilization, demonstrating that HSC mobilization involved inhibition of both, Cxcr4- and Robo4-mediated niche interactions.

### Eph and Ephrins

Expression of Eph (erythropoietin-producing hepatocellular) molecules and their cognate ligands Ephrins on the HSCs and stromal cells has been documented by several studies (Nguyen et al., 2016). EphA1–Eph5 and ephrinA1-ephrin-A5 is expressed by murine HSCs (Ting et al., 2010). Human bone marrow-derived MSCs have been shown to express high levels of EphB1, 2, 4 and ephrin B1, B2, where Eph-ephrin interactions regulate bone remodeling (Nguyen et al., 2016). Several studies have shown that EphB4/ephrin-B2 interaction regulates stromal cell support of HSCs, especially during erythropoiesis (Suenobu et al., 2002; Wang et al., 2002; Foo et al., 2006). CB-derived HSCs have been shown to express EphB4 (Wang et al., 2002; Foo et al., 2006), while its ligand, ephrin-B2, was found to be expressed on MSCs (Okubo et al., 2006; Nguyen et al., 2016). EphB4 expressed by stromal cells modulates ephrin-B2 expression levels and affects transmigration of HSCs underneath the stromal layer (Okubo et al., 2006). Disruption of EphB4/ephrin-B2 interaction with a peptide inhibitor or shRNA specific for EphB4 in the MSCs reduced their HSC-supportive ability *in vitro* (Nguyen et al., 2016). Consistent with these findings, mouse bone marrow-derived MSCs from transgenic EphB4 mice exhibited an enhanced HSC-supportive capacity (Zhao et al., 2006; Nguyen et al., 2016).

Ephrin-A5 has been reported to enhance HSC trafficking and adhesion (Ting et al., 2010) via phosphorylation of paxillin, a downstream effector of integrin function. An intraperitoneal injection of EphA3 protein into mice mobilized the HSCs into the peripheral blood, whereas peptide-mediated blocking of EphA3/ephrin-A5 interactions affected the *in vivo* homing properties of HSC (Ting et al., 2010).

### Esam1

Ooi et al. (2009) identified endothelial cell-selective adhesion molecule 1 (Esam 1) as a novel HSC marker in purified murine HSCs. It mediates homophilic calcium-independent adhesions. Esam1 KO mice did not show severe hematopoietic defect, but their marrow had higher number of HSCs and fewer T cells. *In vitro* these HSCs gave rise to more granulocyte/monocytes and *in vivo* gave a higher T cell: B cell ratio when transplanted into congenic mice. These studies suggest that Esam1 may play role in HSC proliferation and lineage commitment.

### Cytohesin1

Cytohesin1 (CYTH1), a guanine-nucleotide exchange factor for multiple guanosine triphosphate (GTP)–binding protein, forms a complex with integrin β1 and αL to mediate adhesion to ICAM1. However, it was not studied in the context of HSC adhesion. Using a CYTH1 knock out HSCs, Rak et al. (2017) showed that it is critically required for the adhesion of CB-derived HSCs to primary MSCs, fibronectin and ICAM1 and 2. Although these HSCs showed a reduced ability to engraft and give rise to long-term engraftment in NSG mice, the engraftment defect was not complete indicating that other mechanisms could rescue the CYTH1 deficiency. CYTH1-deficient cells also showed reduced integrin β1 activation, suggesting that CYTH1 mediates integrin-dependent functions.

### ICAM-1

Intercellular adhesion molecule 1 (ICAM-1) has been shown to maintain quiescence of HSCs and also their repopulation in the niche (Liu et al., 2018). ICAM-1^–/–^ mice showed expansion of HSC having impaired quiescence and myeloid bias. ICAM-1-deficient HSCs exhibited normal reconstitution capacity during serial transplantation; however, transplantation of wild type HSCs in ICAM-1^–/–^ mice showed that ICAM-1 deficient niche affects HSC quiescence and fails to retain HSCs in the bone marrow. These data revealed the role of ICAM-1 as an important adhesion molecule involved in the retention of HSCs in the bone marrow niche.

### Serum Response Factor (Srf)

The serum response factor (*Srf*), a transcription factor, regulates genes controlling cytoskeletal components involved in cell spreading, adhesion, and migration. *Srf* binds to CC(A/T)6GG (CArG) motif, but its transcriptional activity depends on various signal-regulated cofactors. Ragu et al. (2010) found that Srf plays a role in HSC adhesion. They found that conditional deletion of *Srf* in BM cells expanded HSCs and multipotent progenitors (MPPs), without having much effect on cell cycle dynamics. However, *Srf* loss resulted in defect in mature cell formation and severe thrombocytopenia. *Srf*-null HSCs also showed reduced engraftment properties. *Srf* was found to control the genes governing cell migration and adhesion. *Srf*-null HSCs had impaired expression of the integrin network and decreased adherence *in vitro*. Consistent with this, the *Srf*-null mice had increased numbers of circulating HSCs in their peripheral blood. These studies suggest that *Srf* regulates HSC adhesion through HSC-ECM interactions and integrin signaling.

## Role of Glycosylation Signatures in Functionality of Adhesion Molecules

Selectin binding activity of adhesion molecules expressed on hematopoietic cells and endothelial cells is determined by specific glycoproteins produced by post-translational modifications. These modifications are carried out by the activity of very specific enzymes known as glycosyltransferases. These enzymes link the serine or threonine residues of proteins to oligosaccharides capped with sialyl Lewis × moiety, α2–3 sialylated, α1–3 ucosylated tetrasaccharides (Lowe, 2002). Interaction of L-selectin molecules expressed on hematopoietic cells and their receptors play important role in homing of these cells to the specified tissues, mostly having high endothelial venules (HEVs). Various types of HEVs having hematopoietic cell binding capacities are found in different secondary lymphoid organs (Harlan and Winn, 2002).

Initial *in vitro* experiments showed that the anti- L-selectin antibody, MEL-14 blocked the binding of hematopoietic cells to HEV in frozen tissue sections (Gallatin et al., 1983). The *in vivo* experiments also confirmed that the blocking L-selectin using MEL-14 antibody restricted the HSC homing (Kansas, 1996). The fact that the hematopoietic cells can efficiently bind to the L-selectin receptors which have disrupted peptide epitopes (Stamper and Woodruff, 1977), but not with those treated with sialidase (Rosen et al., 1985, 1989), suggests the involvement of polysaccharides in the binding process. *In vivo* treatment of mice with intra-venous injections of sialidase diminished the *in vivo* homing of HSCs confirming their role in the active binding of these adhesion molecules to their receptors (Rosen et al., 1985, 1989).

Many of the adhesion receptors like selectins and integrins have distinct glycosylation signatures that are considered vital to their roles in homing of HSCs (Levesque and Winkler, 2016). Most of the selectin ligands such as PSGL-1, CD34, CD44, GlyCAM-1, MADCAM-1 that are expressed on HSCs are mucin-type glycoproteins having sialylated, fucosylated glycan chains (Kansas, 1996). Selectins recognize the tetrasaccharide structures like sialyl Lewisx (sLex) displayed at the ends of these long glycan chains or glycolipids, however, purified sLex tetrasaccharide does not completely block the selectin-mediated cell adhesion (Somers et al., 2000). Although most hematopoietic cells express PSGL-1 (CD162), a well-characterized selectin receptor that binds to all three selectins, they can adhere to selectins only when glycosyl-transferases required to generate the sLex tetrasaccharide are co-expressed. In particular, fucosyl-transferase (FucT)-VII is absolutely essential to generate functional selectin receptors. Fut7^–/–^ leukocytes cannot adhere to P- and E-selectin, even though they express PSGL-1 (Malı et al., 1996). Observations made in GlcNAcT-I null as well as PSGL-1 null mice show that these receptors show more active binding with P-selectin compared to E-selectin (Ellies et al., 1998; Yang et al., 1999; Xia et al., 2002). Similarly, sialomucin CD34 which is expressed on primitive human hematopoietic cells and is considered to be a major human HSPC marker is an L-selectin receptor (Baumheter et al., 1993). On the other hand, mice lacking CD34 expression are seen to have normal hematopoietic cell homing activity (Cheng et al., 1996). Parallel to this scenario, CD34 is considered to be the HSPC marker in human system, while it has no definite marking in murine system. These studies also underscore the need to study these adhesion molecules in detail across various animal systems.

Integrins interact with ECM molecules to form complex multi-protein structures, which link the ECM to the actin skeleton, thereby initiating bidirectional signaling. Both ECM molecules and integrins are glycoproteins, and *N*-glycans are known to modulate their conformation and regulate their functions. Great variations in both the number and the distribution of *N*-glycosylation sites are found in α and β subunits of integrins. Especially, the ability of integrins to form functional dimers depends on N-linked oligosaccharides (Janik et al., 2010). Enzymatic removal of sialic acid residues is known to increase the fibronectin binding activity of β1 integrins (Semel et al., 2002). In other studies the *N*-glycosylation was found to be important for pairing of α and β subunits of integrins (Zheng et al., 1994), which is important for acquiring the required conformational changes in these subunits for binding. Interestingly, unnatural N-glycosylation of β1 subunit in the α5β1 integrin increases its active ligand-binding conformation, suggesting the importance of glycosylation in acquiring ligand-binding changes in integrins (Luo et al., 2003). Glycosylation status of various integrins is altered in various cancer cells, and these changes modulate tumorigenesis and metastasis (Von Lampe et al., 1993; Glavey et al., 2015). *N*-glycans are known to play important roles in HSC adhesion and migration by modulating the function of integrins. Thus, it becomes necessary to study the role of glycosylation signatures in the modulation of various HSC properties such as migration, homing and engraftment.

## Other Factors Affecting HSC Adhesion

### Role of Stromal Cell-Derived EVs in HSC Adhesion

Recent literature shows that EVs form one of the important mediators of intercellular communication. Apart from the larger vesicles released by the cells undergoing apoptosis (apoptotic bodies, >1,000 nm in size), EVs are also secreted by healthy cells, either constitutively or after activation. These EVs are classified into two main subtypes, namely, micro-vesicles (MVs, up to ∼1,000 nm in diameter) and exosomes (40–100-nm diameter;Graça and Willem, 2013; Cocucci and Meldolesi, 2015).

Exosomes are formed by fusion of multi-vesicular endosomes (MVE) with the plasma membrane, whereas MVs are shed by outward blebbing of the plasma membrane (Hessvik and Llorente, 2018; Zhang et al., 2019). MVs and exosomes are generally separated by differential ultra-centrifugation of body fluids such as blood, urine etc. or conditioned media of cultured cells and characterized by electron microscopy. It may be noted that smaller MVs (100 nM) are also secreted by many cells and they could interfere in the separation process. Both, MVs and exosomes contain membrane proteins that are known to cluster at the plasma membrane or at endosomes. Tetraspanins such as CD63, CD81, CD82, CD53, and CD37 are the most commonly used identification markers for the purified preparations of EVs, but these markers cannot distinguish between MVs and exosomes. Due to their endosomal origin, exosomes contain endosome-associated proteins, e.g., Rab GTPase, SNAREs, Annexins, and flotillin (van Niel et al., 2006). In spite of the differences in their size and mode of biogenesis both, MVs and exosomes function similarly when released into extra-cellular space by binding and fusing to their target cells. Exosomes are formed in a regulated manner within the cell and being part of the endosome pathway, they are considered more likely to transport adhesion molecules, receptors and other molecules to nearby or distant cells to generate a specific function, as compared to the MVs. However, as compared to exosomes, the mechanism involved in the biogenesis of MVs is not yet completely understood. Also, most studies do not clearly define the origin of the EVs under study, making it difficult to assign the reported effect(s) to either MVs or to exosomes. Making a clear distinction between these two types of EVs could facilitate a better understanding of the role played by each of them. Recent literature shows that EVs form one of the important mediators of crosstalk between the HSCs and their microenvironment. Stromal cell-derived EVs are being studied in the context of HSC biology. These EVs have been shown to mimic the immuno-regulatory property of the parent cells (Kordelas et al., 2014; Amarnath et al., 2015; Wang et al., 2016). Likewise, they have also been shown to have salutary effects on the HSCs in terms of their proliferation, viability, differentiation, protection from irradiation-induced toxicity, rejuvenation etc. (De Luca et al., 2016; Goloviznina et al., 2016; Wen et al., 2016; Xie et al., 2016; Schoefinius et al., 2017; Stik et al., 2017; Kulkarni et al., 2018; Morhayim et al., 2020). MVs (microvesicles) and exosomes, which are the subpopulations of EVs have also been explored for their differential effects on HSC regulation. ESC-derived MVs have been shown to reprogram HSPCs by horizontal transfer of mRNAs and protein (Ratajczak et al., 2006). Similarly, Endothelial Progenitor Cells (EPC)-derived MVs are reported to increase the angiogenesis in the ECs (Deregibus et al., 2010). CB-HSCs when treated with MVs derived from leukemic cell line Jukrat resulted in an increased cell number and viability, and a decrease in p53 tumor suppressor gene expression, as compared to the HSCs treated with normal bone marrow cell-derived EVs (Razmkhah et al., 2015). Exosomes derived from MSCs isolated from bone marrow of multiple myeloma (MM) patients showed a significantly different miRNA and protein content than the exosomes derived from their normal counterparts leading to an enhanced myeloma tumor growth suggesting that the differential secretion of exosomes from bone marrow-derived MSCs under altered conditions (Roccaro et al., 2013). The study of exosomes uptake and their effect on HSCs clearly shows that the HSCs preferential take up exosomes secreted by HSC-supportive cells from the mixture of exosomes from HSC-supportive and HSC non-supportive cells. These exosomes also support the proliferation of co-cultured HSCs and improve their survival by reducing apoptosis levels in them (Stik et al., 2017). Exosomes from acute myeloid leukemia directly repress HSC activity by decreasing clonogenicity, reduced CXCR4 and c-Kit expression and repression of several hematopoietic transcription factors. These exosomes were also shown to increase mobilization of HSCs through down-regulation of SCF and SDF-1α in stromal cells (Huan et al., 2015), hinting at their important role in HSC adhesion and mobilization. However, effects of EVs on the HSC adhesion have not been extensively studied.

Chemotactic migration via CXCR4-SDF1α axis of HSCs to the bone marrow micro-environment and their subsequent adhesion to the stromal cells via various adhesion molecules are crucial pre-requisites in successful transplantation. MSC-derived EVs were shown to up-regulate CXCR4 expression on them leading to their augmented *in vivo* migration to the BM niche in NSG mice (De Luca et al., 2016). However, they did not find any specific miRNA regulating CXCR4, and hence, one can speculate that either CXCR4-specific mRNA was transferred from the EVs to the HSCs or the CXCR4 expression was enhanced by some indirect mechanisms. Using murine system, we have reported that the HSCs co-cultured with micro-vesicles (MVs) of nitric oxide-primed BMSCs (primed MVs) show significantly higher expression of CXCR4, at both, mRNA and protein levels (Jalnapurkar et al., 2019). Since the level of CXCR4-specific mRNA in the primed MVs was similar to control MVs, but that of VEGF-A was significantly higher, this increased expression of CXCR4 could be correlated with the activation of primed MV-mediated VEGFA-eNOS pathway in the HSCs, as CXCR4 has been shown to be a direct target of nitric oxide (Cui et al., 2009). Human MSC-derived MVs have been shown to harbor adhesion molecules such as fibronectin, ezrin, IQGAP1, CD47, integrins etc. (Kim et al., 2012). Likewise, Morhayim et al. (2020) showed that the EVs isolated from fetal calvaria-derived osteoblastic cells contained cell adhesion molecules like EPCAM, ICAM1, and ITG7. Mobilization of HSCs involves disruption of their adhesion to stromal cells. de Kruijf et al. (2018) showed that infusion of MSC-derived EVs resulted in mobilization of HSCs into the blood stream, but the molecular mechanism involved in this process was not studied. Although this study could be taken as an indicative of role of EVs in HSC adhesion, direct effects of EVs on HSC adhesion to stromal cells have not been examined. Such studies would be of immense importance in clinical transplantations. Our lab is actively involved in studying this aspect.

Aging is known to affect the homing and engraftment ability of HSCs (Liang et al., 2005). We have shown that a brief exposure of young HSCs with the EVs secreted by aged MSCs significantly reduces their engraftment by reducing their autophagy-inducing mRNAs (Kulkarni et al., 2018). Consistent with our earlier studies (Singh et al., 2016); we could attribute this to aging-mediated increase in the activation of AKT in the stromal cells. Although, we did not assess the effect of such reduced autophagy on the expression of adhesion molecules on young HSCs, aging seems to be one of the physiological signals altering the HSC adhesion to stroma. Since aging is considered as a consequence of chronic low-grade inflammation (Sanada et al., 2018), it is possible that other inflammatory conditions could also affect the macromolecular composition of stromal EVs and affect HSC adhesion. This aspect needs to be formally examined.

Collectively, the reports suggest that EVs from specific type of niche cells can support HSC survival and maintenance. The HSCs are regulated by EV-mediated cross-talk with the niche and other types of distantly located cells, and alteration in the micro-environment and the resultant altered profile of EVs (MVs or exosomes or their relative balance) produced by niche cells may alter the HSC response and their functionality. Thus, HSC functionality can be modulated by “priming” the parent cells with pharmacological means to modulate their EV profiles for their application for therapeutic purpose (Kale, 2019).

#### EVs Secreted by Hematopoietic Cells and Their Effect on HSC Adhesion

Besides the cells of non-hematopoietic origin, HSC niche consists of hematopoietic cells like megakaryocytes (Mgk) (Bruns et al., 2014; Zhao et al., 2014) and macrophages (Winkler et al., 2010) that affect HSC fate. Since almost all cells secrete EVs, the EVs secreted by Mgk and macrophages could also affect HSC adhesion. Winkler et al. (2010) have shown that marrow macrophages maintain the HSCs niche and their removal leads to HSC mobilization, suggesting that macrophages are involved in HSC adhesion to stroma. Likewise, Mgk are required for expansion of osteoblasts after irradiation, and thus, indirectly regulate HSC engraftment (Olson et al., 2013). Whether macrophages and Mgk participate in HSC adhesion to stroma via the EVs secreted by them needs to be examined. Zhang et al. (2020) have suggested that the EVs secreted by aging immune cells may differ in their molecular content. However, in this study the effect of such altered EVs on HSCs was not examined. It will be interesting to see whether the EVs of aging immune cells affect adhesion molecule profile of the HSCs.

### Effect of Aging on HSC Adhesion

In specific conditions such as aging, HSCs show a reduced expression of CXCR4 (Shao et al., 2011) and consequent reduced response to the chemokines (Akunuru and Geiger, 2016). Adherence ability of HSCs to stromal cells is also known to be compromised during aging. Aged mice show fivefold increase in mobilized HSC numbers confirming their diminished adhesive properties (Xing et al., 2006), which could be due to decreased levels of SDF-1α in their bone marrow (Tuljapurkar et al., 2011). Further, the compromised property of aged bone marrow niche to retain HSCs was also documented earlier; wherein the young HSCs when transplanted to aged recipients demonstrated reduced frequency of homing, as compared to those transplanted to young recipients (Liang et al., 2005). Aged bone marrow changes from an osteoblast-dominant to an adipocyte-dominant one. This change in the microenvironment coupled with the reduced bone density might be the reason for the aging-mediated decrease in the HSC functionality. The studies aiming at understanding the changes in aged BM physiology and attempts to modulate it could help us in finding the treatment for various malignant and non-malignant gerontological hematopoietic diseases.

## Orchestration of HSC Adhesion Mechanisms in Homing and Engraftment of HSCs

Homing of HSCs to the appropriate place in the bone marrow niche is an important process involving coordinated functioning of various signaling and adhesion molecules. Individual roles of most of the adhesion molecules have been discussed earlier in this review. However, these molecules do not operate independently, but rather work in an orchestrated manner to guide the infused HSCs to their exact destination in the bone marrow. The processes of migration and homing require sequential formation and disruption of various adhesion bonds between the HSCs and the bone marrow cells. The important steps involve the rolling of HSCs on the walls of endothelial capillaries, followed by their further migration into the bone marrow compartment and finally their engraftment in the specific HSC-supportive microenvironment.

Bone marrow is a primary organ of hematopoiesis and large numbers of hematopoietic cells are known to traffic from and to the bone marrow. Thus, for efficient homing of HSCs to the bone marrow very specific mechanisms are required to selectively attract the HSCs to the bone marrow niche. First, the intravenously infused HSCs migrate to the bone marrow niche via chemotactic migration mediated by the SDF1α-CXCR4 axis. Thereafter, the L- and P- selectin molecules expressed on the ECs present on the lining of the blood sinusoids and capillaries in bone marrow get involved in the homing process, as the ligands for these selectins are expressed on the HSCs (Sahin and Buitenhuis, 2012). Once brought in the close proximity of the ECs, HSCs interact with them and the process of initial tethering and rolling along the walls of endothelial capillaries begins. The specificity of this process can be underscored by the observation that only the bone marrow sinusoids and capillaries support the rolling process of HSCs on their endothelial linings and such process is not supported by the adjoining bone vessels (Mazo et al., 1998). Further, as mentioned before, only E- and P-selectins are involved in this initial process of HSC rolling, whereas L- selectin does not play any role in this process. Another selectin-independent mechanism of the HSC rolling involves the interaction of α4 integrin with VCAM-1 molecules expressed on ECs (Mazo et al., 1998).

Once adhered to the endothelial lining, HSCs undergo *trans*-endothelial migration to migrate along the SDF-1 gradient. E- and P- selectins once again play an important role in the recruitment of HSC to the ECs and subsequent *trans*-endothelial migration (Frenette et al., 1998; Naiyer et al., 1999). HSCs are known to express high levels of α4β7 integrins, which drive the adhesion of HSCs to the ECs to initiate the migration. HSCs knocked out for α4β7 integrins show reduced expression of CXCR4 – the chemokine receptor for SDF-1, and show reduced ability to migrate and home to bone marrow niche. Lethal irradiation is known to induce the mucosal addressin cell adhesion molecule-1 (MAdCAM-1), the ligand for α4β7 integrin, on the bone marrow ECs. Expression of MAdCAM-1on ECs promotes the increased adhesion of HSCs to the endothelium, resulting in their successful homing and engraftment (Murakami et al., 2016).

The integrin molecules further direct the homing of the LT-HSCs. Integrins exhibit a special phenomenon in that they need to acquire the specific conformationally activated state to bind to their ligands. Various types of soluble extracellular molecules such as cytokines, cations, etc. acting at different concentrations, help in the formation of the various conformations of integrins having different affinity for the specific ligands expressed on the neighboring cells. Divalent cation binding sites are present near the ligand binding site on the integrin molecules, thereby allowing the conformational flexibility for binding. Studies show that the Mg^2+^ at a concentration of about 1mM initiates β1 integrin activation in the absence of any cytokines, while the Ca^2+^ concentration of more than 10mM inhibits β1 integrin activation by cytokines. The site of active bone resorption near the osteoclasts has normally more than 10 mM concentration of Ca^2+^ making it an inhabitable place for HSC adherence and homing. On the other hand, Mn^2+^ which is known to be the super-activator of β1 integrin is found at high concentration in compact bones niches, making it a favorable place to home for the HSCs (Levesque and Winkler, 2016).

## Future Perspective

The importance of adhesion mechanisms in the success of clinical transplantation evoked an immense interest in the adhesion mechanisms involved in the HSC-niche interactions. Several studies have identified various types of adhesion molecules expressed on the HSCs and also on their niche cells. Most studies have used genetic or antibody-/peptide-mediated function-blocking approach. However, very few studies have revealed the physiological cues initiating these mechanisms. If these cues could be identified they would not only improve the efficacy of HSC transplantations, but might also contribute toward the regenerative medicine as well.

The role of EVs in the HSC adhesion needs to be investigated in greater details. Since EVs could form “ready-to-use” biologic, identification of their role in HSC fate determination may accrue clinical benefits.

Notably, none of the studies have looked at adhesion mechanisms operative within the stromal cell population. During steady-state conditions the stromal cells may not interact with each other, however, it is quite possible that under the influence of physiological cues like hypoxia, inflammation etc. and also in response to the secretome of HSCs receiving differentiation signals (e.g., EPO), the stromal cells also could initiate homotypic (e.g., MSCs to MSCs) or heterotypic (e.g., MSCs to osteoblasts or ECs) interactions among themselves and these interactions could in turn lead to HSC adhesion and fate change. This possibility also needs to be addressed. Such studies could provide better insight to the mechanisms involved in HSC adhesion.

## Author Contributions

RK and VK researched the literature and wrote the manuscript. VK conceived the contents, wrote, edited, and approved the final version. Both authors contributed to the article and approved the submitted version.

## Conflict of Interest

The authors declare that the research was conducted in the absence of any commercial or financial relationships that could be construed as a potential conflict of interest.

## References

[B1] Akunuru S. Geiger H. (2016). Aging, clonality, and rejuvenation of hematopoietic stem cells. *Trends Mol. Med.* 22 701–712. 10.1016/j.molmed.2016.06.003 27380967 PMC4969095

[B2] Amarnath S. Foley J. E. Farthing D. E. Gress R. E. Laurence A. Eckhaus M. A. (2015). Bone marrow-derived mesenchymal stromal cells harness purinergenic signaling to tolerize human T h1 cells in vivo. *Stem Cells* 33 1200–1212. 10.1002/stem.1934 25532725 PMC4376558

[B3] Arai F. Hirao A. Ohmura M. Sato H. Matsuoka S. Takubo K. (2004). Tie2/angiopoietin-1 signaling regulates hematopoietic stem cell quiescence in the bone marrow niche. *Cell* 118 149–161. 10.1016/j.cell.2004.07.004 15260986

[B4] Arai F. Hosokawa K. Toyama H. Matsumoto Y. Suda T. (2012). Role of N-cadherin in the regulation of hematopoietic stem cells in the bone marrow niche. *Ann. N. Y. Acad. Sci.* 1266 72–77. 10.1111/j.1749-6632.2012.06576.x 22901259

[B5] Askmyr M. Sims N. A. Martin T. J. Purton L. E. (2009). What is the true nature of the osteoblastic hematopoietic stem cell niche? *Trends Endocrinol. Metab.* 20 303–309. 10.1016/j.tem.2009.03.004 19595609

[B6] Avigdor A. Goichberg P. Shivtiel S. Dar A. Peled A. Samira S. (2004). CD44 and hyaluronic acid cooperate with SDF-1 in the trafficking of human CD34+ stem/progenitor cells to bone marrow. *Blood* 103 2981–2989. 10.1182/blood-2003-10-3611 15070674

[B7] Azizidoost S. Bavarsad M. S. Bavarsad M. S. Shahrabi S. Jaseb K. Rahim F. (2015). The role of notch signaling in bone marrow niche. *Hematology* 20 93–103. 10.1179/1607845414y.0000000167 24724873

[B8] Barczyk M. Carracedo S. Gullberg D. (2010). Integrins. *Cell Tissue Res.* 339:269. 10.1007/s00441-009-0834-6 PMC2784866 19693543

[B9] Baron M. H. (2003). Embryonic origins of mammalian hematopoiesis. *Exp. Hematol.* 31 1160–1169. 10.1016/j.exphem.2003.08.019 14662321

[B10] Baumheter S. Singer M. S. Henzel W. Hemmerich S. Renz M. Rosen S. D. (1993). Binding of L-selectin to the vascular sialomucin CD34. *Science* 262 436–438. 10.1126/science.7692600 7692600

[B11] Beerman I. Luis T. C. Singbrant S. Celso C. L. Méndez-Ferrer S. (2017). The evolving view of the hematopoietic stem cell niche. *Exp. Hematol.* 50 22–26. 10.1016/j.exphem.2017.01.008 28189651 PMC5466495

[B12] Bellucci R. De Propris M. S. Buccisano F. Lisci A. Leone G. Tabilio A. (1999). Modulation of VLA-4 and L-selectin expression on normal CD34+ cells during mobilization with G-CSF. *Bone Marrow Trans.* 23 1–8. 10.1038/sj.bmt.1701522 10037043

[B13] Bigas A. Espinosa L. (2012). Hematopoietic stem cells: to be or Notch to be. *Blood J. Am. Soc. Hematol.* 119 3226–3235. 10.1182/blood-2011-10-355826 22308291

[B14] Bonig H. Priestley G. V. Papayannopoulou T. (2006). Hierarchy of molecular-pathway usage in bone marrow homing and its shift by cytokines. *Blood* 107, 79–86. 10.1182/blood-2005-05-2023 16141352 PMC1895342

[B15] Bourke V. Watchman C. Dieudonne A. Bolch W. (2009). The spatial profile of blood vessels and hematopoietic stem cells within the marrow cavities of the human skeleton. *J. Nucl. Med.* 50(Suppl. 2), 269–269. 10.1182/blood-2008-12-192922 PMC2774549 19749092

[B16] Bromberg O. Frisch B. J. Weber J. M. Porter R. L. Civitelli R. Calvi L. M. (2012). Osteoblastic N-cadherin is not required for microenvironmental support and regulation of hematopoietic stem and progenitor cells. *Blood J. Am. Soc. Hematol.* 120 303–313. 10.1182/blood-2011-09-377853 22596259 PMC3398755

[B17] Bruns I. Lucas D. Pinho S. Ahmed J. Lambert M. P. Kunisaki Y. (2014). Megakaryocytes regulate hematopoietic stem cell quiescence through CXCL4 secretion. *Nat. Med.* 20:1315. 10.1038/nm.3707 25326802 PMC4258871

[B18] Butko E. Pouget C. Traver D. (2016). Complex regulation of HSC emergence by the Notch signaling pathway. *Dev. Biol.* 409 129–138. 10.1016/j.ydbio.2015.11.008 26586199 PMC4708088

[B19] Butler J. M. Nolan D. J. Vertes E. L. Varnum-Finney B. Kobayashi H. Hooper A. T. (2010). Endothelial cells are essential for the self-renewal and repopulation of Notch-dependent hematopoietic stem cells. *Cell Stem Cell* 6 251–264. 10.1016/j.stem.2010.02.001 20207228 PMC2866527

[B20] Butler J. T. Abdelhamed S. Kurre P. (2018). Extracellular vesicles in the hematopoietic microenvironment. *Haematologica* 103 382–394. 10.3324/haematol.2017.183335 29439185 PMC5830368

[B21] Calvi L. M. Adams G. B. Weibrecht K. W. Weber J. M. Olson D. P. Knight M. C. (2003). Osteoblastic cells regulate the haematopoietic stem cell niche. *Nature* 425 841–846. 10.1038/nature02040 14574413

[B22] Cao H. Heazlewood S. Y. Williams B. Cardozo D. Nigro J. Oteiza A. (2016). The role of CD44 in fetal and adult hematopoietic stem cell regulation. *Haematologica* 101 26–37. 10.3324/haematol.2015.135921 26546504 PMC4697889

[B23] Cecyn K. Z. Kimura E. Lima D. M. S. Yamamoto M. Bordin J. O. de Oliveira J. S. R. (2018). Expression of adhesion molecules on CD34+ cells from steady-state bone marrow before and after mobilization and their association with the yield of CD34+ cells. *Blood Res.* 53 61–70. 29662864 10.5045/br.2018.53.1.61 PMC5898996

[B24] Chambers S. M. Shaw C. A. Gatza C. Fisk C. J. Donehower L. A. Goodell M. A. (2007). Aging hematopoietic stem cells decline in function and exhibit epigenetic dysregulation. *PLoS Biol.* 5:e201. 10.1371/journal.pbio.0050201 17676974 PMC1925137

[B25] Chen M. Geng J. G. (2006). P-selectin mediates adhesion of leukocytes, platelets, and cancer cells in inflammation, thrombosis, and cancer growth and metastasis. *Arch. Immunol. Ther. Exp.* 54 75–84. 10.1007/s00005-006-0010-6 16648968

[B26] Cheng J. Baumhueter S. Cacalano G. Carver-Moore K. Thibodeaux H. Thomas R. (1996). Hematopoietic defects in mice lacking the sialomucin CD34. *Blood* 87 479–490. 10.1182/blood.v87.2.479.bloodjournal872479 8555469

[B27] Chitteti B. R. Kacena M. A. Voytik-Harbin S. L. Srour E. F. (2015). Modulation of hematopoietic progenitor cell fate in vitro by varying collagen oligomer matrix stiffness in the presence or absence of osteoblasts. *J. Immunol. Methods* 425 108–113. 10.1016/j.jim.2015.07.001 26159389

[B28] Choi J. S. Harley B. A. (2017). Marrow-inspired matrix cues rapidly affect early fate decisions of hematopoietic stem and progenitor cells. *Sci. Adv.* 3:e1600455. 10.1126/sciadv.1600455 28070554 PMC5218514

[B29] Choi J. S. Mahadik B. P. Harley B. A. (2015). Engineering the hematopoietic stem cell niche: Frontiers in biomaterial science. *Biotechnol. J.* 10 1529–1545. 10.1002/biot.201400758 26356030 PMC4724421

[B30] Chotinantakul K. Leeanansaksiri W. (2012). Hematopoietic stem cell development, niches, and signaling pathways. *Bone Marrow Res.* 2012:270425. 10.1155/2012/270425 PMC3413998 22900188

[B31] Ciriza J. García-Ojeda M. E. (2010). Expression of migration-related genes is progressively upregulated in murine Lineage-Sca-1+ c-Kit+ population from the fetal to adult stages of development. *Stem Cell Res. Ther* 1:14. 10.1186/scrt14 20637061 PMC2905090

[B32] Cocucci E. Meldolesi J. (2015). Ectosomes and exosomes:shedding the confusion between extracellular vesicles. *Trends Cell Biol.* 25 364–372. 10.1016/j.tcb.2015.01.004 25683921

[B33] Corselli M. Chin C. J. Parekh C. Sahaghian A. Wang W. Ge S. (2013). Perivascular support of human hematopoietic stem/progenitor cells. *Blood J. Am. Soc. Hematol.* 121 2891–2901. 10.1182/blood-2012-08-451864 PMC3707421 23412095

[B34] Cui X. Chen J. Zacharek A. Roberts C. Yang Y. Chopp M. (2009). Nitric oxide donor up-regulation of SDF1/CXCR4 and Ang1/Tie2 promotes neuroblast cell migration after stroke. *J. Neurosci. Res.* 87 86–95. 10.1002/jnr.21836 18711749 PMC2606920

[B35] Dar A. Kollet O. Lapidot T. (2006). Mutual, reciprocal SDF-1/CXCR4 interactions between hematopoietic and bone marrow stromal cells regulate human stem cell migration and development in NOD/SCID chimeric mice. *Exp. Hematol.* 34 967–975. 10.1016/j.exphem.2006.04.002 16863903

[B36] de Kruijf E. J. F. Zuijderduijn R. Stip M. C. Fibbe W. E. van Pel M. (2018). Mesenchymal stromal cells induce a permissive state in the bone marrow that enhances G-CSF-induced hematopoietic stem cell mobilization in mice. *Exp. Hematol.* 64 59–70. 29775645 10.1016/j.exphem.2018.05.002

[B37] De Luca L. Trino S. Laurenzana I. Simeon V. Calice G. Raimondo S. (2016). MiRNAs and piRNAs from bone marrow mesenchymal stem cell extracellular vesicles induce cell survival and inhibit cell differentiation of cord blood hematopoietic stem cells: a new insight in transplantation. *Oncotarget* 7:6676. 10.18632/oncotarget.6791 26760763 PMC4872742

[B38] Deregibus M. C. Tetta C. Camussi G. (2010). The dynamic stem cell microenvironment is orchestrated by microvesicle-mediated transfer of genetic information. *Histol. Histopathol.* 25 397–404. 20054810 10.14670/HH-25.397

[B39] Dimitroff C. J. Lee J. Y. Fuhlbrigge R. C. Sackstein R. (2000). A distinct glycoform of CD44 is an L-selectin ligand on human hematopoietic cells. *Proc. Natl. Acad. Sci. U.S.A.* 97 13841–13846. 10.1073/pnas.250484797 11095749 PMC17663

[B40] Dimitroff C. J. Lee J. Y. Schor K. S. Sandmaier B. M. Sackstein R. (2001). Differential L-selectin binding activities of human hematopoietic cell L-selectin ligands. HCELL and PSGL-1. *J. Biol. Chem.* 276 47623–47631. 10.1074/jbc.m105997200 11591704

[B41] Eliceiri B. P. (2001). Integrin and growth factor receptor crosstalk. *Circ. Res.* 89 1104–1110. 10.1161/hh2401.101084 11739274

[B42] Ellies L. G. Tsuboi S. Petryniak B. Lowe J. B. Fukuda M. Marth J. D. (1998). Core 2 oligosaccharide biosynthesis distinguishes between selectin ligands essential for leukocyte homing and inflammation. *Immunity* 9 881–890. 10.1016/s1074-7613(00)80653-6 9881978

[B43] Eto T. Winkler I. Purton L. E. Lévesque J. P. (2005). Contrasting effects of P-selectin and E-selectin on the differentiation of murine hematopoietic progenitor cells. *Exp. Hematol.* 33 232–242. 10.1016/j.exphem.2004.10.018 15676218

[B44] Fernandez L. Rodriguez S. Huang H. Chora A. Fernandes J. Mumaw C. (2008). Tumor necrosis factor-α and endothelial cells modulate Notch signaling in the bone marrow microenvironment during inflammation. *Exp. Hematol.* 36 545–558. 18439488 10.1016/j.exphem.2007.12.012 PMC3437760

[B45] Flaumenhaft R. Dilks J. R. Rozenvayn N. Monahan-Earley R. A. Feng D. Dvorak A. M. (2005). The actin cytoskeleton differentially regulates platelet α-granule and dense-granule secretion. *Blood* 105 3879–3887. 10.1182/blood-2004-04-1392 15671445

[B46] Foo S. S. Turner C. J. Adams S. Compagni A. Aubyn D. Kogata N. (2006). Ephrin-B2 controls cell motility and adhesion during blood-vessel-wall assembly. *Cell* 124 161–173. 10.1016/j.cell.2005.10.034 16413489

[B47] Frenette P. S. Mayadas T. N. Rayburn H. Hynes R. O. Wagner D. D. (1996). Susceptibility to infection and altered hematopoiesis in mice deficient in both P-and E-selectins. *Cell* 84 563–574. 10.1016/s0092-8674(00)81032-6 8598043

[B48] Frenette P. S. Subbarao S. Mazo I. B. Von Andrian U. H. Wagner D. D. (1998). Endothelial selectins and vascular cell adhesion molecule-1 promote hematopoietic progenitor homing to bone marrow. *Proc. Natl. Acad. Sci. U.S.A.* 95 14423–14428. 10.1073/pnas.95.24.14423 9826716 PMC24389

[B49] Gallatin W. M. Weissman I. L. Butcher E. C. (1983). A cell-surface molecule involved in organ-specific homing of lymphocytes. *Nature* 304 30–34. 10.1038/304030a0 6866086

[B50] Gawaz M. Langer H. May A. E. (2005). Platelets in inflammation and atherogenesis. *J. Clin. Invest.* 115 3378–3384. 10.1172/jci27196 16322783 PMC1297269

[B51] Gekas C. Graf T. (2013). CD41 expression marks myeloid-biased adult hematopoietic stem cells and increases with age. *Blood J. Am. Soc. Hematol.* 121 4463–4472. 10.1182/blood-2012-09-457929 23564910

[B52] Glavey S. V. Huynh D. Reagan M. R. Manier S. Moschetta M. Kawano Y. (2015). The cancer glycome: carbohydrates as mediators of metastasis. *Blood Rev.* 29 269–279. 10.1016/j.blre.2015.01.003 25636501

[B53] Goloviznina N. A. Verghese S. C. me Yoon Y. Taratula O. Marks D. L. Kurre P. (2016). Mesenchymal stromal cell-derived extracellular vesicles promote myeloid-biased multipotent hematopoietic progenitor expansion via toll-like receptor engagement. *J. Biol. Chem.* 291 24607–24617. 10.1074/jbc.m116.745653 27758863 PMC5114412

[B54] Gong J. K. (1978). Endosteal marrow: a rich source of hematopoietic stem cells. *Science* 199 1443–1445. 10.1126/science.75570 75570

[B55] Graça R. Willem S. (2013). Extracellular vesicles: exosomes, microvesicles, and friends. *J. Cell Biol.* 4 373–383. 10.1083/jcb.201211138 23420871 PMC3575529

[B56] Greenbaum A. Hsu Y. M. Day R. B. Schuettpelz L. G. Christopher M. J. Borgerding J. N. (2013). CXCL12 in early mesenchymal progenitors is required for haematopoietic stem-cell maintenance. *Nature* 495 227–230. 10.1038/nature11926 23434756 PMC3600148

[B57] Greenbaum A. M. Revollo L. D. Woloszynek J. R. Civitelli R. Link D. C. (2012). N-cadherin in osteolineage cells is not required for maintenance of hematopoietic stem cells. *Blood J. Am. Soc. Hematol.* 120 295–302. 10.1182/blood-2011-09-377457 22323481 PMC3398761

[B58] Guezguez B. Campbell C. J. Boyd A. L. Karanu F. Casado F. L. Di Cresce C. (2013). Regional localization within the bone marrow influences the functional capacity of human HSCs. *Cell Stem Cell* 13 175–189. 10.1016/j.stem.2013.06.015 23910084

[B59] Gunji Y. Nakamura M. Hagiwara T. Hayakawa K. Matsushita H. Osawa H. (1992). Expression and function of adhesion molecules on human hematopoietic stem cells: CD34+ LFA-1- cells are more primitive than CD34+ LFA-1+ cells. *Blood* 80 429–436. 10.1182/blood.v80.2.429.429 1378320

[B60] Haas S. Hansson J. Klimmeck D. Loeffler D. Velten L. Uckelmann H. (2015). Inflammation-induced emergency megakaryopoiesis driven by hematopoietic stem cell-like megakaryocyte progenitors. *Cell Stem Cell* 17 422–434. 10.1016/j.stem.2015.07.007 26299573

[B61] Hahne M. Jäger U. Isenmann S. Hallmann R. Vestweber D. (1993). Five tumor necrosis factor-inducible cell adhesion mechanisms on the surface of mouse endothelioma cells mediate the binding of leukocytes. *J. Cell Biol.* 121 655–664. 10.1083/jcb.121.3.655 7683689 PMC2119562

[B62] Haines N. Irvine K. D. (2003). Glycosylation regulates Notch signalling. *Nat. Rev. Mol. Cell Biol.* 4 786–797. 10.1038/nrm1228 14570055

[B63] Harlan J. M. Winn R. K. (2002). Leukocyte-endothelial interactions: clinical trials of anti-adhesion therapy. *Crit. Care Med.* 30 S214–S219. 12004238 10.1097/00003246-200205001-00007

[B64] Haug J. S. He X. C. Grindley J. C. Wunderlich J. P. Gaudenz K. Ross J. T. (2008). N-cadherin expression level distinguishes reserved versus primed states of hematopoietic stem cells. *Cell Stem Cell* 2 367–379. 10.1016/j.stem.2008.01.017 18397756

[B65] Hessvik N. P. Llorente A. (2018). Current knowledge on exosome biogenesis and release. *Cell Mol. Life Sci.* 75 193–208. 10.1007/s00018-017-2595-9 28733901 PMC5756260

[B66] Hill R. Wu H. (2009). PTEN, stem cells, and cancer stem cells. *J. Biol. Chem.* 284 11755–11759. 10.1074/jbc.r800071200 19117948 PMC2673242

[B67] Hirsch E. Iglesias A. Potocnik A. J. Hartmann U. Fässler R. (1996). Impaired migration but not differentiation of haematopoietic stem cells in the absence of β 1 integrins. *Nature* 380 171–175. 10.1038/380171a0 8600394

[B68] Hosokawa K. Arai F. Yoshihara H. Iwasaki H. Hembree M. Yin T. (2010a). Cadherin-based adhesion is a potential target for niche manipulation to protect hematopoietic stem cells in adult bone marrow. *Cell Stem Cell* 6 194–198. 10.1016/j.stem.2009.04.013 20207221

[B69] Hosokawa K. Arai F. Yoshihara H. Iwasaki H. Nakamura Y. Gomei Y. (2010b). Knockdown of N-cadherin suppresses the long-term engraftment of hematopoietic stem cells. *Blood J. Am. Soc. Hematol.* 116 554–563. 10.1182/blood-2009-05-224857 20427705

[B70] Hosokawa K. Arai F. Yoshihara H. Nakamura Y. Gomei Y. Iwasaki H. (2007). Function of oxidative stress in the regulation of hematopoietic stem cell-niche interaction. *Biochem. Biophys. Res. Commun.* 363 578–583. 10.1016/j.bbrc.2007.09.014 17897629

[B71] Huan J. Hornick N. I. Goloviznina N. A. Kamimae-Lanning A. N. David L. L. Wilmarth P. A. (2015). Coordinate regulation of residual bone marrow function by paracrine trafficking of AML exosomes. *Leukemia* 29 2285–2295. 10.1038/leu.2015.163 26108689 PMC4834971

[B72] Huang J. C. Lu Y. Sidorova J. Chien S. Oehler V. Kopecky K. J. (2016). Age-Related Changes in the Interaction of Acute Myeloid Leukemia with the Bone Marrow Microenvironment Correlate with Response to Treatment and Survival: A Role for L-Selectin. *Blood* 128 5063–5063. 10.1182/blood.v128.22.5063.5063

[B73] Hynes R. O. (2002). Integrins: bidirectional, allosteric signaling machines. *Cell* 110 673–687. 12297042 10.1016/s0092-8674(02)00971-6

[B74] Jacobsen S. E. W. (2005). Defining ‘stemness’: Notch and Wnt join forces? *Nat. Immunol.* 6 234–236. 10.1038/ni0305-234 15716971

[B75] Jalnapurkar S. Moirangthem R. D. Singh S. Limaye L. Kale V. (2019). Microvesicles secreted by nitric oxide-primed mesenchymal stromal cells boost the engraftment potential of hematopoietic stem cells. *Stem Cells* 37 128–138. 10.1002/stem.2912 30290030

[B76] Janik M. E. Lityñska A. Vereecken P. (2010). Cell migration—the role of integrin glycosylation. *Biochim. Biophys. Acta* 1800 545–555. 10.1016/j.bbagen.2010.03.013 20332015

[B77] Kale V. P. (2019). Application of “Primed” mesenchymal stromal cells in hematopoietic stem cell transplantation: current status and future prospects. *Stem Cells Dev* 28 1473–1479. 10.1089/scd.2019.0149 31559908

[B78] Kansas G. S. (1996). Selectins and their ligands: current concepts and controversies. *Blood* 88 3259–3287. 10.1182/blood.v88.9.3259.bloodjournal8893259 8896391

[B79] Kansas G. S. Pavalko F. M. (1996). The cytoplasmic domains of E-and P-selectin do not constitutively interact with alpha-actinin and are not essential for leukocyte adhesion. *J. Immunol.* 157 321–325. 8683133

[B80] Karpova D. Ritchey J. Holt M. Monlish D. Schuettpelz L. G. Yang W. (2016). Expansion and Maintenance of Hematopoietic Stem and Progenitor Cells in Course of Long-Term Inhibition of CXCR4/CXCL12 Signaling. *Blood* 128:2648 10.1182/blood.v128.22.2648.2648

[B81] Katayama Y. Hidalgo A. Peired A. Frenette P. S. (2004). Integrin α4β7 and its counterreceptor MAdCAM-1 contribute to hematopoietic progenitor recruitment into bone marrow following transplantation. *Blood* 104 2020–2026. 10.1182/blood-2003-12-4157 15161666

[B82] Khurana S. Schouteden S. Manesia J. K. Santamaria-Martínez A. Huelsken J. Lacy-Hulbert A. (2016). Outside-in integrin signalling regulates haematopoietic stem cell function via Periostin-Itgav axis. *Nat. Commun.* 7 1–14. 10.1038/ncomms13500 PMC5146274 27905395

[B83] Kiel M. J. Acar M. Radice G. L. Morrison S. J. (2009). Hematopoietic stem cells do not depend on N-cadherin to regulate their maintenance. *Cell Stem Cell* 4 170–179. 10.1016/j.stem.2008.10.005 19119091 PMC2681089

[B84] Kiel M. J. Radice G. L. Morrison S. J. (2007). Lack of evidence that hematopoietic stem cells depend on N-cadherin-mediated adhesion to osteoblasts for their maintenance. *Cell Stem Cell* 1 204–217. 10.1016/j.stem.2007.06.001 18371351

[B85] Kiel M. J. Yilmaz ÖH. Iwashita T. Yilmaz O. H. Terhorst C. Morrison S. J. (2005). SLAM family receptors distinguish hematopoietic stem and progenitor cells and reveal endothelial niches for stem cells. *Cell* 121 1109–1121. 10.1016/j.cell.2005.05.026 15989959

[B86] Kim H. S. Choi D. Y. Yun S. J. Choi S. M. Kang J. W. Jung J. W. (2012). Proteomic analysis of microvesicles derived from human mesenchymal stem cells. *J. Proteome Res.* 11 839–849. 10.1021/pr200682z 22148876

[B87] Kimble J. E. White J. G. (1981). On the control of germ cell development in Caenorhabditis elegans. *Dev. Biol.* 81 208–219. 10.1016/0012-1606(81)90284-0 7202837

[B88] Koenig J. M. Baron S. Luo D. Benson N. A. Deisseroth A. B. (1999). L-selectin expression enhances clonogenesis of CD34+ cord blood progenitors. *Pediatr. Res.* 45 867–870. 10.1203/00006450-199906000-00015 10367780

[B89] Kopan R. (2012). Notch Signaling. *Cold Spring Harb. Perspect. Biol.* 4 a011213. 10.1101/cshperspect.a011213 23028119 PMC3475170

[B90] Kopan R. Ilagan M. X. G. (2009). The Canonical Notch Signaling Pathway: Unfolding the Activation Mechanism. *Cell* 137 216–233. 10.1016/j.cell.2009.03.045 19379690 PMC2827930

[B91] Kordelas L. Rebmann V. Ludwig A. K. Radtke S. Ruesing J. Doeppner T. R. (2014). MSC-derived exosomes: a novel tool to treat therapy-refractory graft-versus-host disease. *Leukemia* 28 970–973. 10.1038/leu.2014.41 24445866

[B92] Krause D. S. Lazarides K. Lewis J. B. von Andrian U. H. Van Etten R. A. (2014). Selectins and their ligands are required for homing and engraftment of BCR-ABL1+ leukemic stem cells in the bone marrow niche. *Blood J. Am. Soc. Hematol.* 123 1361–1371. 10.1182/blood-2013-11-538694 24394666 PMC3938148

[B93] Kulkarni R. Bajaj M. Ghode S. Jalnapurkar S. Limaye L. Kale V. P. (2018). Intercellular transfer of microvesicles from young mesenchymal stromal cells rejuvenates aged murine hematopoietic stem cells. *Stem Cells* 36 420–433. 10.1002/stem.2756 29230885

[B94] Lee-Thedieck C. Rauch N. Fiammengo R. Klein G. Spatz J. P. (2012). Impact of substrate elasticity on human hematopoietic stem and progenitor cell adhesion and motility. *J. Cell Sci.* 125 3765–3775. 10.1242/jcs.095596 22553208

[B95] Leiva M. Quintana J. A. Ligos J. M. Hidalgo A. (2016). Haematopoietic ESL-1 enables stem cell proliferation in the bone marrow by limiting TGFβ availability. *Nat. Commun.* 7 1–10. 10.1038/ncomms10222 PMC4729861 26742601

[B96] Levesque J. P. (2012). N (o)-cadherin role for HSCs. *Blood J. Am. Soc. Hematol.* 120 237–238. 10.1182/blood-2012-05-431148 22791770

[B97] Levesque J. P. Winkler I. G. (2016). Cell adhesion molecules in normal and malignant hematopoiesis: from bench to bedside. *Curr. Stem Cell Rep.* 2 356–367. 10.1007/s40778-016-0066-0

[B98] Lévesque J. P. Zannettino A. C. Pudney M. Niutta S. Haylock D. N. Snapp K. R. (1999). PSGL-1-mediated adhesion of human hematopoietic progenitors to P-selectin results in suppression of hematopoiesis. *Immunity* 11 369–378. 10.1016/s1074-7613(00)80112-0 10514015

[B99] Li P. Zon L. I. (2010). Resolving the controversy about N-cadherin and hematopoietic stem cells. *Cell Stem Cell* 6 199–202. 10.1016/j.stem.2010.02.007 20207222

[B100] Liang Y. Van Zant G. Szilvassy S. J. (2005). Effects of aging on the homing and engraftment of murine hematopoietic stem and progenitor cells. *Blood* 106 1479–1487. 10.1182/blood-2004-11-4282 15827136 PMC1895199

[B101] Liu P. Xu B. Hock C. E. Nagele R. Sun F. F. Wong P. Y. (1998). NO modulates P-selectin and ICAM-1 mRNA expression and hemodynamic alterations in hepatic I/R. *Am. J. Physiol. Heart Circ. Physiol.* 275 H2191–H2198. 10.1152/ajpheart.1998.275.6.H2191 9843819

[B102] Liu Y. F. Zhang S. Y. Chen Y. Y. Shi K. Zou B. Liu J. (2018). ICAM-1 deficiency in the bone marrow niche impairs quiescence and repopulation of hematopoietic stem cells. *Stem Cell Reports.* 11, 258–273. 10.1016/j.stemcr.2018.05.016 29937143 PMC6117479

[B103] Loeffler D. Wang W. Hopf A. Hilsenbeck O. Bourgine P. E. Rudolf F. (2018). Mouse and human HSPC immobilization in liquid culture by CD43-or CD44-antibody coating. *Blood J. Am. Soc. Hematol.* 131 1425–1429. 10.1182/blood-2017-07-794131 29453290

[B104] Lord B. I. Testa N. G. Hendry J. H. (1975). The relative spatial distributions of CFUs and CFUc in the normal mouse femur. *Blood* 46 65–72. 10.1182/blood.v46.1.65.65 1131427

[B105] Lowe J. B. (2002). Glycosylation in the control of selectin counter-receptor structure and function. *Immunol. Rev.* 186 19–36. 10.1034/j.1600-065x.2002.18603.x 12234359

[B106] Luo B. H. Springer T. A. Takagi J. (2003). Stabilizing the open conformation of the integrin headpiece with a glycan wedge increases affinity for ligand. *Proc. Natl. Acad. Sci. U.S.A.* 100 2403–2408. 10.1073/pnas.0438060100 12604783 PMC151353

[B107] Malı P. Thall A. D. Petryniak B. Rogers C. E. Smith P. L. Marks R. M. (1996). The α (1, 3) fucosyltransferase Fuc-TVII controls leukocyte trafficking through an essential role in L-, E-, and P-selectin ligand biosynthesis. *Cell* 86 643–653. 10.1016/s0092-8674(00)80137-3 8752218

[B108] Mazo I. B. Gutierrez-Ramos J. C. Frenette P. S. Hynes R. O. Wagner D. D. Von Andrian U. H. (1998). Hematopoietic progenitor cell rolling in bone marrow microvessels: parallel contributions by endothelial selectins and vascular cell adhesion molecule 1. *J. Exp. Med.* 188 465–474. 10.1084/jem.188.3.465 9687524 PMC2212463

[B109] Mendez-Ferrer S. Frenette P. S. (2007). Hematopoietic stem cell trafficking: regulated adhesion and attraction to bone marrow microenvironment. *Ann. N. Y. Acad. Sci.* 1116 392–413. 10.1196/annals.1402.086 18083941

[B110] Mitjavila-Garcia M. T. Cailleret M. Godin I. Nogueira M. M. Cohen-Solal K. Schiavon V. (2002). Expression of CD41 on hematopoietic progenitors derived from embryonic hematopoietic cells. *Development* 129 2003–2013. 11934866 10.1242/dev.129.8.2003

[B111] Möhle R. Murea S. Kirsch M. Haas R. (1995). Differential expression of L-selectin, VLA-4, and LFA-1 on CD34+ progenitor cells from bone marrow and peripheral blood during G-CSF-enhanced recovery. *Exp. Hematol.* 23 1535–1542. 8542944

[B112] Moirangthem R. D. Singh S. Adsul A. Jalnapurkar S. Limaye L. Kale V. P. (2015). Hypoxic niche-mediated regeneration of hematopoiesis in the engraftment window is dominantly affected by oxygen tension in the milieu. *Stem Cells Dev.* 24 2423–2436. 10.1089/scd.2015.0112 26107807 PMC4599134

[B113] Morhayim J. Ghebes C. A. Erkeland S. J. ter Borg M. N. Hoogenboezem R. M. Bindels E. M. (2020). Identification of osteolineage cell-derived extracellular vesicle cargo implicated in hematopoietic support. *FASEB J.* 34 5435–5452. 10.1096/fj.201902610r 32086861 PMC7136136

[B114] Murakami J. L. Xu B. Franco C. B. Hu X. Galli S. J. Weissman I. L. (2016). Evidence that β7 integrin regulates hematopoietic stem cell homing and engraftment through interaction with MAdCAM-1. *Stem Cells Dev.* 25 18–26. 10.1089/scd.2014.0551 26422691 PMC4692116

[B115] Nagasawa T. Hirota S. Tachibana K. Takakura N. Nishikawa S. I. Kitamura Y. (1996). Defects of B-cell lymphopoiesis and bone-marrow myelopoiesis in mice lacking the CXC chemokine PBSF/SDF-1. *Nature* 382 635–638. 10.1038/382635a0 8757135

[B116] Naiyer A. J. Jo D. Y. Ahn J. Mohle R. Peichev M. Lam G. (1999). Stromal Derived Factor-1–Induced Chemokinesis of Cord Blood CD34+ Cells (Long-Term Culture-Initiating Cells) Through Endothelial Cells Is Mediated by E-Selectin. *Blood J. Am. Soc. Hematol.* 94 4011–4019. 10.1182/blood.v94.12.4011.424k10_4011_4019 10590044

[B117] Nguyen T. M. Arthur A. Gronthos S. (2016). The role of Eph/ephrin molecules in stromal–hematopoietic interactions. *Int. J. Hematol.* 103 145–154. 10.1007/s12185-015-1886-x 26475284

[B118] Nie Y. Han Y. C. Zou Y. R. (2008). CXCR4 is required for the quiescence of primitive hematopoietic cells. *J. Exp. Med.* 205 777–783. 10.1084/jem.20072513 18378795 PMC2292218

[B119] Nilsson S. K. Johnston H. M. Coverdale J. A. (2001). Spatial localization of transplanted hemopoietic stem cells: inferences for the localization of stem cell niches. *Blood J. Am. Soc. Hematol.* 97 2293–2299. 10.1182/blood.v97.8.2293 11290590

[B120] Notta F. Doulatov S. Laurenti E. Poeppl A. Jurisica I. Dick J. E. (2011). Isolation of single human hematopoietic stem cells capable of long-term multilineage engraftment. *Science* 333 218–221. 10.1126/science.1201219 21737740

[B121] Ohishi K. Varnum-Finney B. Bernstein I. D. (2002). The notch pathway: modulation of cell fate decisions in hematopoiesis. *Int. J. Hematol.* 75 449–459. 10.1007/bf02982106 12095143

[B122] Okubo T. Yanai N. Obinata M. (2006). Stromal cells modulate ephrinB2 expression and transmigration of hematopoietic cells. *Exp. Hematol.* 34 330–338. 10.1016/j.exphem.2005.12.003 16543067

[B123] Olson T. S. Caselli A. Otsuru S. Hofmann T. J. Williams R. Paolucci P. (2013). Megakaryocytes promote murine osteoblastic HSC niche expansion and stem cell engraftment after radioablative conditioning. *Blood J. Am. Soc. Hematol.* 121 5238–5249. 10.1182/blood-2012-10-463414 23667055 PMC3695366

[B124] Ooi A. L. Karsunky H. Majeti R. Butz S. Vestweber D. Ishida T. (2009). The adhesion molecule esam1 is a novel hematopoietic stem cell marker. *Stem Cells* 27 653–661. 10.1634/stemcells.2008-0824 19074415 PMC4186794

[B125] Peled A. Kollet O. Ponomaryov T. Petit I. Franitza S. Grabovsky V. (2000). The chemokine SDF-1 activates the integrins LFA-1, VLA-4, and VLA-5 on immature human CD34+ cells: role in transendothelial/stromal migration and engraftment of NOD/SCID mice. *Blood J. Am. Soc. Hematol.* 95 3289–3296. 10.1182/blood.v95.11.3289.011k33_3289_3296 10828007

[B126] Pello O. M. del Carmen Moreno-Ortiz M. Rodriìguez-Frade J. M. Martiìnez-MunÞoz L. Lucas D. Gómez L. (2006). SOCS up-regulation mobilizes autologous stem cells through CXCR4 blockade. *Blood* 108 3928–3937. 10.1182/blood-2006-02-006353 16912231

[B127] Pinho S. Frenette P. S. (2019). Haematopoietic stem cell activity and interactions with the niche. *Nat. Rev. Mol. Cell Biol.* 20 303–320. 10.1038/s41580-019-0103-9 30745579 PMC6483843

[B128] Potocnik A. J. Brakebusch C. Fässler R. (2000). Fetal and adult hematopoietic stem cells require β1 integrin function for colonizing fetal liver, spleen, and bone marrow. *Immunity* 12 653–663. 10.1016/s1074-7613(00)80216-2 10894165

[B129] Poulos M. G. Guo P. Kofler N. M. Pinho S. Gutkin M. C. Tikhonova A. (2013). Endothelial Jagged-1 is necessary for homeostatic and regenerative hematopoiesis. *Cell Rep.* 4 1022–1034. 10.1016/j.celrep.2013.07.048 24012753 PMC3805263

[B130] Puch S. Armeanu S. Kibler C. Johnson K. R. Muller C. A. Wheelock M. J. (2001). N-cadherin is developmentally regulated and functionally involved in early hematopoietic cell differentiation. *J. Cell Sci.* 114 1567–1577. 11282032 10.1242/jcs.114.8.1567

[B131] Ragu C. Elain G. Mylonas E. Ottolenghi C. Cagnard N. Daegelen D. (2010). The transcription factor Srf regulates hematopoietic stem cell adhesion. *Blood J. Am. Soc. Hematol.* 116 4464–4473. 10.1182/blood-2009-11-251587 20709909

[B132] Rak J. Foster K. Potrzebowska K. Talkhoncheh M. S. Miharada N. Komorowska K. (2017). Cytohesin 1 regulates homing and engraftment of human hematopoietic stem and progenitor cells. *Blood J. Am. Soc. Hematol.* 129 950–958. 10.1182/blood-2016-06-720649 27899358 PMC5335807

[B133] Ratajczak J. Miekus K. Kucia M. Zhang J. Reca R. Dvorak P. (2006). Embryonic stem cell-derived microvesicles reprogram hematopoietic progenitors: evidence for horizontal transfer of mRNA and protein delivery. *Leukemia* 20 847–856. 10.1038/sj.leu.2404132 16453000

[B134] Razmkhah F. Soleimani M. Mehrabani D. Karimi M. H. Kafi-Abad S. A. (2015). Leukemia cell microvesicles promote survival in umbilical cord blood hematopoietic stem cells. *EXCLI J.* 14 423. 10.17179/excli2015-101 PMC4743482 26862318

[B135] Reichert D. Friedrichs J. Ritter S. Käubler T. Werner C. Bornhäuser M. (2015). Phenotypic, morphological and adhesive differences of human hematopoietic progenitor cells cultured on murine versus human mesenchymal stromal cells. *Sci. Rep.* 5 1–14. 10.1038/srep15680 PMC4620509 26498381

[B136] Roccaro A. M. Sacco A. Maiso P. Azab A. K. Tai Y. T. Reagan M. (2013). BM mesenchymal stromal cell–derived exosomes facilitate multiple myeloma progression. *J. Clin. Invest.* 123 1542–1555. 10.1172/jci66517 23454749 PMC3613927

[B137] Rosen S. D. Chi S. I. True D. D. Singer M. S. Yednock T. A. (1989). Intravenously injected sialidase inactivates attachment sites for lymphocytes on high endothelial venules. *J. Immunol.* 142 1895–1902. 2921520

[B138] Rosen S. D. Singer M. S. Yednock T. A. Stoolman L. M. (1985). Involvement of sialic acid on endothelial cells in organ-specific lymphocyte recirculation. *Science* 228 1005–1007. 10.1126/science.4001928 4001928

[B139] Sahin A. O. Buitenhuis M. (2012). Molecular mechanisms underlying adhesion and migration of hematopoietic stem cells. *Cell Adhesion Migrat.* 6 39–48. 10.4161/cam.18975 22647939 PMC3364136

[B140] Sanada F. Taniyama Y. Muratsu J. Otsu R. Shimizu H. Rakugi H. (2018). Source of chronic inflammation in aging. *Front. Cardiovasc. Med.* 5:12. 10.3389/fcvm.2018.00012 29564335 PMC5850851

[B141] Sasnoor L. M. Kale V. P. Limaye L. S. (2003). Supplementation of conventional freezing medium with a combination of catalase and trehalose results in better protection of surface molecules and functionality of hematopoietic cells. *J. Hematother. Stem Cell Res.* 12 553–564. 10.1089/152581603322448268 14594512

[B142] Schoefinius J. S. Brunswig-Spickenheier B. Speiseder T. Krebs S. Just U. Lange C. (2017). Mesenchymal stromal cell-derived extracellular vesicles provide long-term survival after total body irradiation without additional hematopoietic stem cell support. *Stem Cells* 35 2379–2389. 10.1002/stem.2716 29024236

[B143] Schofield R. (1978). The relationship between the spleen colony-forming cell and the haemopoietic stem cell. *Blood Cells* 4 7–25. 747780

[B144] Semel A. C. Seales E. C. Singhal A. Eklund E. A. Colley K. J. Bellis S. L. (2002). Hyposialylation of integrins stimulates the activity of myeloid fibronectin receptors. *J. Biol. Chem.* 277 32830–32836. 10.1074/jbc.m202493200 12091385

[B145] Shao H. Xu Q. Wu Q. Ma Q. Salgueiro L. Wang J. A. (2011). Defective CXCR4 expression in aged bone marrow cells impairs vascular regeneration. *J. Cell Mol. Med.* 15 2046–2056. 10.1111/j.1582-4934.2010.01231.x 21143386 PMC3076550

[B146] Shimizu Y. Van Seventer G. A. Horgan K. J. Shaw S. (1990). Costimulation of proliferative responses of resting CD4+ T cells by the interaction of VLA-4 and VLA-5 with fibronectin or VLA-6 with laminin. *J. Immunol.* 145 59–67. 1972721

[B147] Singh S. Moirangthem R. D. Vaidya A. Jalnapurkar S. Limaye L. Kale V. (2016). AKT signaling prevailing in mesenchymal stromal cells modulates the functionality of hematopoietic stem cells via intercellular communication. *Stem Cells* 34 2354–2367. 10.1002/stem.2409 27300259

[B148] Smith-Berdan S. Nguyen A. Hassanein D. Zimmer M. Ugarte F. Ciriza J. (2011). Robo4 cooperates with CXCR4 to specify hematopoietic stem cell localization to bone marrow niches. *Cell Stem Cell* 8 72–83. 10.1016/j.stem.2010.11.030 21211783 PMC3625377

[B149] Somers W. S. Tang J. Shaw G. D. Camphausen R. T. (2000). Insights into the molecular basis of leukocyte tethering and rolling revealed by structures of P-and E-selectin bound to SLeX and PSGL-1. *Cell* 103 467–479. 10.1016/s0092-8674(00)00138-0 11081633

[B150] Sprinzak D. Lakhanpal A. Lebon L. Santat L. A. Fontes M. E. Anderson G. A. (2010). Cis-interactions between Notch and Delta generate mutually exclusive signalling states. *Nature* 465 86–90. 10.1038/nature08959 20418862 PMC2886601

[B151] Stamper H. B. Woodruff J. J. (1977). An in vitro model of lymphocyte homing: I. Characterization of the interaction between thoracic duct lymphocytes and specialized high-endothelial venules of lymph nodes. *J. Immunol.* 119 772–780. 407305

[B152] Stik G. Crequit S. Petit L. Durant J. Charbord P. Jaffredo T. (2017) Extracellular vesicles of stromal origin target and support hematopoietic stem and progenitor cells. *J. Cell Biol*. 216, 2217–2230. 10.1083/jcb.201601109 28630143 PMC5496607

[B153] Suenobu S. Takakura N. Inada T. Yamada Y. Yuasa H. Zhang X. Q. (2002). A role of EphB4 receptor and its ligand, ephrin-B2, in erythropoiesis. *Biochem. Biophys. Res. Commun.* 293 1124–1131. 10.1016/s0006-291x(02)00330-3 12051776

[B154] Sugiyama T. Kohara H. Noda M. Nagasawa T. (2006). Maintenance of the hematopoietic stem cell pool by CXCL12-CXCR4 chemokine signaling in bone marrow stromal cell niches. *Immunity* 25 977–988. 10.1016/j.immuni.2006.10.016 17174120

[B155] Szmigielska-Kaplon A. Szemraj J. Hamara K. Robak M. Wolska A. Pluta A. (2014). Polymorphism of CD44 influences the efficacy of CD34+ cells mobilization in patients with hematological malignancies. *Biol. Blood Marrow Transplant.* 20 986–991. 10.1016/j.bbmt.2014.03.019 24680978

[B156] Takada Y. Ye X. Simon S. (2007). The integrins. *Genome Biol.* 8:215. 10.1186/gb-2007-8-5-215 PMC1929136 17543136

[B157] Ting M. J. Day B. W. Spanevello M. D. Boyd A. W. (2010). Activation of ephrin A proteins influences hematopoietic stem cell adhesion and trafficking patterns. *Exp. Hematol.* 38 1087–1098. 10.1016/j.exphem.2010.07.007 20655977

[B158] Tuljapurkar S. R. McGuire T. R. Brusnahan S. K. Jackson J. D. Garvin K. L. Kessinger M. A. (2011). Changes in human bone marrow fat content associated with changes in hematopoietic stem cell numbers and cytokine levels with aging. *J. Anat.* 219 574–581. 10.1111/j.1469-7580.2011.01423.x 21923862 PMC3191309

[B159] Umemoto T. Yamato M. Ishihara J. Shiratsuchi Y. Utsumi M. Morita Y. (2012). Integrin-αvβ3 regulates thrombopoietin-mediated maintenance of hematopoietic stem cells. *Blood J. Am. Soc. Hematol.* 119 83–94. 10.1182/blood-2011-02-335430 22096247 PMC3251239

[B160] van Niel G. Porto-Carreiro I. Simoes S. Raposo G. (2006). Exosomes: a common pathway for a specialized function. *J. Biochem.* 40 13–21. 10.1093/jb/mvj128 16877764

[B161] Varnum-Finney B. Xu L. Brashem-Stein C. Nourigat C. Flowers D. Bakkour S. (2000). Pluripotent, cytokine-dependent, hematopoietic stem cells are immortalized by constitutive Notch1 signaling. *Nat. Med.* 6 1278–1281. 10.1038/81390 11062542

[B162] Vestweber D. Blanks J. E. (1999). Mechanisms that regulate the function of the selectins and their ligands. *Physiol. Rev.* 79, 181–213. 10.1152/physrev.1999.79.1.181 9922371

[B163] Von Lampe B. Stallmach A. Riecken E. O. (1993). Altered glycosylation of integrin adhesion molecules in colorectal cancer cells and decreased adhesion to the extracellular matrix. *Gut* 34 829–836. 10.1136/gut.34.6.829 8314518 PMC1374271

[B164] Wagers A. J. Allsopp R. C. Weissman I. L. (2002). Changes in integrin expression are associated with altered homing properties of Lin-/loThy1. 1loSca-1+ c-kit+ hematopoietic stem cells following mobilization by cyclophosphamide/granulocyte colony-stimulating factor. *Exp. Hematol.* 30 176–185. 10.1016/s0301-472x(01)00777-9 11823053

[B165] Wagner W. Wein F. Roderburg C. Saffrich R. Diehlmann A. Eckstein V. (2008). Adhesion of human hematopoietic progenitor cells to mesenchymal stromal cells involves CD44. *Cells Tissues Organs* 188 160–169. 10.1159/000112821 18160820

[B166] Wang L. Gu Z. Zhao X. Yang N. Wang F. Deng A. (2016). Extracellular vesicles released from human umbilical cord-derived mesenchymal stromal cells prevent life-threatening acute graft-versus-host disease in a mouse model of allogeneic hematopoietic stem cell transplantation. *Stem Cells Dev.* 25, 1874–1883. 10.1089/scd.2016.0107 27649744

[B167] Wang L. D. Wagers A. J. (2011). Dynamic niches in the origination and differentiation of haematopoietic stem cells. *Nat. Rev. Mol. Cell Biol.* 12 643–655. 10.1038/nrm3184 21886187 PMC4040463

[B168] Wang W. Yu S. Myers J. Wang Y. Xin W. W. Albakri M. (2017). Notch2 blockade enhances hematopoietic stem cell mobilization and homing. *Haematologica* 102 1785–1795. 10.3324/haematol.2017.168674 28729299 PMC5622863

[B169] Wang Y. Zhang L. Li Y. Chen L. Wang X. Guo W. (2015). Exosomes/microvesicles from induced pluripotent stem cells deliver cardioprotective miRNAs and prevent cardiomyocyte apoptosis in the ischemic myocardium. *Int. J. Cardiol.* 192 61–69. 10.1016/j.ijcard.2015.05.020 26000464 PMC4469495

[B170] Wang W. Yu S. Zimmerman G. Wang Y. Myers J. Yu V. W. (2015). Notch Receptor-Ligand Engagement Maintains Hematopoietic Stem Cell Quiescence and Niche Retention. *Stem Cells* 33 2280–2293. 10.1002/stem.2031 25851125 PMC4478168

[B171] Wang Z. Miura N. Bonelli A. Mole P. Carlesso N. Olson D. P. (2002). Receptor tyrosine kinase, EphB4 (HTK), accelerates differentiation of select human hematopoietic cells. *Blood* 99, 2740–2747. 10.1182/blood.v99.8.2740 11929761

[B172] Watt S. M. Forde S. P. (2008). The central role of the chemokine receptor, CXCR4, in haemopoietic stem cell transplantation: will CXCR4 antagonists contribute to the treatment of blood disorders? *Vox Sanguinis* 94 18–32. 18042197 10.1111/j.1423-0410.2007.00995.x

[B173] Wein F. Pietsch L. Saffrich R. Wuchter P. Walenda T. Bork S. (2010). N-cadherin is expressed on human hematopoietic progenitor cells and mediates interaction with human mesenchymal stromal cells. *Stem Cell Res.* 4 129–139. 10.1016/j.scr.2009.12.004 20116358

[B174] Wen S. Dooner M. Cheng Y. Papa E. Del Tatto M. Pereira M. (2016). Mesenchymal stromal cell-derived extracellular vesicles rescue radiation damage to murine marrow hematopoietic cells. *Leukemia* 30 2221–2231. 10.1038/leu.2016.107 27150009 PMC5093052

[B175] Wierenga P. K. Weersing E. Dontje B. de Haan G. van Os R. (2006). Differential role for very late antigen-5 in mobilization and homing of hematopoietic stem cells. *Bone Marrow Transplant.* 38 789–797. 10.1038/sj.bmt.1705534 17086206

[B176] Wilson A. Murphy M. J. Oskarsson T. Kaloulis K. Bettess M. D. Oser G. M. (2004). c-Myc controls the balance between hematopoietic stem cell self-renewal and differentiation. *Genes Dev.* 18 2747–2763. 10.1101/gad.313104 15545632 PMC528895

[B177] Wilson A. Radtke F. (2006). Multiple functions of Notch signaling in self-renewing organs and cancer. *FEBS Lett.* 580 2860–2868. 10.1016/j.febslet.2006.03.024 16574107

[B178] Winkler I. G. Barbier V. Nowlan B. Jacobsen R. N. Forristal C. E. Patton J. T. (2012). Vascular niche E-selectin regulates hematopoietic stem cell dormancy, self renewal and chemoresistance. *Nat. Med.* 18:1651. 10.1038/nm.2969 23086476

[B179] Winkler I. G. Sims N. A. Pettit A. R. Barbier V. Nowlan B. Helwani F. (2010). Bone marrow macrophages maintain hematopoietic stem cell (HSC) niches and their depletion mobilizes HSCs. *Blood J. Am. Soc. Hematol.* 116 4815–4828. 10.1182/blood-2009-11-253534 20713966

[B180] Wolfenson H. Lavelin I. Geiger B. (2013). Dynamic regulation of the structure, and functions of integrin adhesions. *Dev. Cell* 24 447–458. 10.1016/j.devcel.2013.02.012 23484852 PMC3878073

[B181] Xia L. Sperandio M. Yago T. McDaniel J. M. Cummings R. D. Pearson-White S. (2002). P-selectin glycoprotein ligand-1–deficient mice have impaired leukocyte tethering to E-selectin under flow. *J. Clin. Invest.* 109 939–950. 10.1172/jci0214151 11927621 PMC150926

[B182] Xie H. Sun L. Zhang L. Liu T. Chen L. Zhao A. (2016). Mesenchymal stem cell-derived microvesicles support ex vivo expansion of cord blood-derived CD34. *Stem Cells Int.* 2016:6493241. 10.1155/2016/6493241 PMC4799819 27042183

[B183] Xie T. Spradling A. C. (2000). A niche maintaining germ line stem cells in the Drosophila ovary. *Science* 290 328–330. 10.1126/science.290.5490.328 11030649

[B184] Xing Z. Ryan M. A. Daria D. Nattamai K. J. Van Zant G. Wang L. (2006). Increased hematopoietic stem cell mobilization in aged mice. *Blood* 108 2190–2197. 10.1182/blood-2005-12-010272 16741255 PMC1895568

[B185] Yamada T. Yamazaki H. Yamane T. Yoshino M. Okuyama H. Tsuneto M. (2003). Regulation of osteoclast development by Notch signaling directed to osteoclast precursors and through stromal cells. *Blood J. Am. Soc. Hematol.* 101 2227–2234. 10.1182/blood-2002-06-1740 12411305

[B186] Yang J. Hirata T. Croce K. Merrill-Skoloff G. Tchernychev B. Williams E. (1999). Targeted gene disruption demonstrates that P-selectin glycoprotein ligand 1 (PSGL-1) is required for P-selectin–mediated but not E-selectin–mediated neutrophil rolling and migration. *J. Exp. Med.* 190 1769–1782. 10.1084/jem.190.12.1769 10601352 PMC2195714

[B187] Zhang J. Niu C. Ye L. Huang H. He X. Tong W. G. (2003). Identification of the haematopoietic stem cell niche and control of the niche size. *Nature* 425 836–841. 10.1038/nature02041 14574412

[B188] Zhang X. Hubal M. Kraus V. B. (2020). Immune cell extracellular vesicles and their mitochondrial content decline with ageing. *Immun. Ageing* 17 1–11. 31911808 10.1186/s12979-019-0172-9 PMC6942666

[B189] Zhang Y. Liu Y. Liu H. Tang W. H. (2019). Exosomes: biogenesis, biologic function and clinical potential. *Cell Biosci* 9:19. 10.1186/s13578-019-0282-2 PMC6377728 30815248

[B190] Zhao C. Irie N. Takada Y. Shimoda K. Miyamoto T. Nishiwaki T. (2006). Bidirectional ephrinB2-EphB4 signaling controls bone homeostasis. *Cell Metab.* 4 111–121. 10.1016/j.cmet.2006.05.012 16890539

[B191] Zhao J. Tang N. Wu K. Dai W. Ye C. Shi J. (2014). MiR-21 simultaneously regulates ERK1 signaling in HSC activation and hepatocyte EMT in hepatic fibrosis. *PLoS One* 9:e8005. 10.1371/journal.pone.0108005 25303175 PMC4193742

[B192] Zhao M. Tao F. Venkatraman A. Li Z. Smith S. E. Unruh J. (2019). N-cadherin-expressing bone and marrow stromal progenitor cells maintain reserve hematopoietic stem cells. *Cell Rep.* 26 652–669. 30650358 10.1016/j.celrep.2018.12.093 PMC6890378

[B193] Zheng M. Fang H. Hakomori S. I. (1994). Functional role of N-glycosylation in alpha 5 beta 1 integrin receptor. De-N-glycosylation induces dissociation or altered association of alpha 5 and beta 1 subunits and concomitant loss of fibronectin binding activity. *J. Biol. Chem.* 269 12325–12331. 7512965

[B194] Zou Y. R. Kottmann A. H. Kuroda M. Taniuchi I. Littman D. R. (1998). Function of the chemokine receptor CXCR4 in haematopoiesis and in cerebellar development. *Nature* 393 595–599. 10.1038/31269 9634238

